# Nutrition claims influence expectations about food attributes, attenuate activity in reward‐associated brain regions during tasting, but do not impact pleasantness

**DOI:** 10.1002/brb3.2828

**Published:** 2022-12-13

**Authors:** Qëndresa Rramani, Youssef Barakat, George Jacob, Kathrin Ohla, Shirley Xue Li Lim, Doris Schicker, Jessica Freiherr, Elodie Saruco, Burkhard Pleger, Bernd Weber, Johannes Schultz

**Affiliations:** ^1^ Center for Economics and Neuroscience (CENs) University of Bonn Bonn Germany; ^2^ Institute of Experimental Epileptology and Cognition Research (IEECR) University of Bonn Bonn Germany; ^3^ NutriAct‐Competence Cluster Nutrition Research Berlin‐Potsdam Nuthetal Germany; ^4^ Firmenich SA Satigny Switzerland; ^5^ Cognitive Neuroscience (INM‐3), Institute of Neuroscience and Medicine Research Center Jülich Germany; ^6^ Sensory Analytics & Technologies Fraunhofer Institute for Process Engineering and Packaging IVV Freising Germany; ^7^ Department of Psychiatry and Psychotherapy Friedrich‐Alexander‐Universität Erlangen‐Nürnberg Erlangen Germany; ^8^ Department of Neurology BG University Clinic Bergmannsheil, Ruhr‐University Bochum Bochum Germany

**Keywords:** expectations, fMRI, food attributes, nutrition claims, taste pleasantness perception, valuation

## Abstract

**Introduction:**

Nutrition claims are one of the most common tools used to improve food decisions. Previous research has shown that nutrition claims impact expectations; however, their effects on perceived pleasantness, valuation, and their neural correlates are not well understood. These claims may have both intended and unintended effects on food perception and valuation, which may compromise their effect on food decisions.

**Methods:**

We investigated the effects of nutrition claims on expectations, perceptions, and valuation of milk‐mix drinks in a behavioral (*n* = 110) and an fMRI (*n* = 39) study. In the behavioral study, we assessed the effects of a “fat‐reduced” and a “protein‐rich” nutrition claim on expected and perceived food attributes of otherwise equal food products. In the fMRI study, we investigated the effect of a “protein‐rich” claim on taste pleasantness perception and valuation, and on their neural correlates during tasting and swallowing.

**Results:**

We found that both nutrition claims increased expected and perceived healthiness and decreased expected but not perceived taste pleasantness. The “protein‐rich” claim increased expected but not perceived satiating quality ratings, while the “fat‐reduced” claim decreased both expected and perceived satiating quality ratings. In the absence *vs*. presence of the “protein‐rich” claim, we observed an increased activity in a cluster extending to the left nucleus accumbens during tasting and an increased functional connectivity between this cluster and a cluster in right middle frontal gyrus during swallowing.

**Conclusion:**

Altogether, we found that nutrition claims impacted expectations and attenuated reward‐related responses during tasting but did not negatively affect perceived pleasantness. Our findings support highlighting the presence of nutrients with positive associations and exposure to foods with nutrition claims to increase their acceptance. Our study offers insights that may be valuable in designing and optimizing the use of nutrition claims.

## 1 INTRODUCTION

Increasing rates of obesity across all age groups and all around the world have deemed the understanding of eating behavior and more particularly food‐related decisions to be an important global health issue (World Health Organization, 2021). Considering the complexity and the burden of obesity and related conditions, public health policies have become increasingly invested in prevention strategies (Gearhardt et al., 2012; Gortmaker et al., 2011; Lyn et al., 2019; Malik et al., 2013). Among the most common strategies in this regard is promoting healthy eating by providing more accessible information on the nutritional and health value of foods in the form of nutrition labels and claims. On one hand, the presence of nutrition labels has been shown to help consumers identify and choose healthier alternatives (Cecchini & Warin, 2016; Hawkes et al., 2015; Hersey et al., 2013; Williams, 2005). On the other hand, different nutrition and health claims have also been shown to have unintended effects on expectations, perceptions, and consumption experience—which has raised the concern that such marketing strategies may often perpetuate unhealthy eating patterns (Chandon & Wansink, 2012; Cornil et al., 2022). Understanding the effects and the mechanisms through which these effects are exerted is thus crucial in optimizing the use of nutrition labels and claims as marketing strategies for food items.

Nutrition claims indicate the presence, absence, and/or level of a certain nutrient in a food product. These claims are particularly interesting when used in novel foods, where marketing strategies may have an exceptionally important impact on consumers' acceptance of these products. The effects of nutrition claims on food preference and choice are not completely understood, although it is supported that they in general increase the expected and perceived healthiness of food products (Nobrega et al., 2020; Oostenbach et al., 2019; Prada et al., 2021; van Trijp & van der Lans, 2007; Williams, 2005). In this context, nutrition claims are unique in the sense that they provide information about the contents of a food product, *i.e*., basic attributes, and also elicit expectations about more abstract attributes, such as the healthiness of a food product (Rangel, 2013). While an enhanced healthiness awareness may motivate consumers to make healthier choices (Chan et al., 2005; Hare et al., 2011; Sonnenberg et al., 2013), it may also compromise expectations of other attribute qualities (known as “health halo” effects^1^). For instance, it has been shown that participants consume more of the same food when labeled as “low‐fat” (*vs*. conventional), possibly due to modulated expectations and perception of healthiness and satiating quality (Belei et al., 2012; Chan et al., 2005; Wansink & Chandon, 2006). Similar effects have been reported regarding taste pleasantness, where the presence of a claim indicating lower fat content has been shown to decrease the expected, and even, although not always, perceived taste pleasantness (Levin & Gaeth, 1988; Ng et al., 2011; Norton et al., 2013; Okamoto & Dan, 2013; Piqueras‐Fiszman & Spence, 2015). This is especially important since experienced taste pleasantness is among the most important determinants of future decisions upon encounter with the same or similar food products (Mela, 2001; Piqueras‐Fiszman & Spence, 2015; Rangel, 2013).

Taste pleasantness has been shown to be affected by several external contexts (Grabenhorst et al., 2008; Piqueras‐Fiszman & Spence, 2015; Plassmann et al., 2008; Schmidt et al., 2017; Spence, 2015). Such contexts have been shown to modulate not only behavioral preference but also activity in brain regions associated with taste processing and taste pleasantness perception (Grabenhorst et al., 2008; Ng et al., 2011; Piqueras‐Fiszman & Spence, 2015). For instance, Grabenhorst et al. (2008) found that perceived taste pleasantness of the same solution differed depending on whether that solution was presented as “monosodium‐glutamate,” “rich and delicious taste,” “rich and delicious flavor,” or “boiled vegetable water.” These cognitive‐level manipulations modulated activity in regions associated with taste and reward processing such as the pregenual cingulate cortex, ventral striatum (vS), and orbitofrontal cortex (OFC) (Grabenhorst et al., 2008). Similarly, perceived taste pleasantness and its neural representation can be enhanced by cues such as price (Plassmann et al., 2008; Schmidt et al., 2017), familiar brands (McClure et al., 2004), and labeling (Enax et al., 2015b; Sörqvist et al., 2013). Such effects on perceived pleasantness are argued to rely on the expectations that these cues elicit (Okamoto & Dan, 2013; Plassmann & Weber, 2015). In this context, Schmidt et al. (2017) found that activity in the brain valuation system (vS, ventromedial prefrontal cortex–vmPFC) was higher when anticipating the same wine presented as more expensive than when presented as less expensive; these differences in the activity of the brain valuation system during anticipation were related to differences in brain activity during taste valuation of the same wines.

Whether and how expectations relate to perception in the context of nutrition claims remains to be investigated. Nutrition claims may elicit different expectations about several attributes, which may, in turn, have different impacts on perceived taste pleasantness, valuation, and choice. For instance, highlighting the healthiness of a food product may negatively impact expected and perceived taste pleasantness; *i.e*., tasty food is often considered to be less healthy and vice versa (so‐called unhealthy‐tasty intuition; see Raghunathan et al., 2006). In line with this, we have previously shown that highlighting healthiness aspects of food via salient labeling increases the weight of healthiness and decreases the weight of taste pleasantness in the decision process via attentional shifts (Enax et al., 2016; Rramani et al., 2020). Moreover, it has been shown that directing attention to healthiness of food via overt instructions (Hare et al., 2011) or via salient labeling (Enax et al., 2015a) increases the behavioral and neural correlates of healthy food items’ value. These effects were linked to an increased connectivity between vmPFC and dorsolateral prefrontal cortex (dlPFC), which may reflect the integration of healthiness in the valuation process. However, whether healthiness expectations about a food product modulate perceived taste pleasantness, valuation, and their neural correlates remains unclear.

Considering that cues may direct attention to aspects of foods such as healthiness (Enax et al., 2016; Rramani et al., 2020), which may impact valuation (Enax et al., 2015a; Hare et al., 2011) and perceived taste pleasantness (Grabenhorst & Rolls, 2008; van Rijn et al., 2018), we hypothesized that nutrition claims may (i) influence expectations about food attributes, (ii) modulate perceived taste pleasantness, and (iii) impact valuation of food. We tested these hypotheses in a behavioral (Study 1) and an fMRI study (Study 2). In Study 1, we assessed how nutrition claims affect expectations and perceptions of taste pleasantness, healthiness, and satiating quality. Moreover, we compared the effects of a claim that emphasizes reduction of a negative attribute (“fat‐reduced”) with those of a claim emphasizing the increase of a positive attribute (“protein‐rich”). In Study 2, we tested whether nutrition claims modulate perceived taste pleasantness and valuation at the behavioral and at the neural level. Specifically, we tested whether activity in brain regions associated with taste pleasantness and valuation (vmPFC, vS, dlPFC, OFC) during tasting and swallowing is modulated by the presence of nutrition claims. Since in Study 1 we did not find different effects of both claims on pleasantness, in Study 2 we only tested one claim. More specifically, we only tested the “protein‐rich” claim, considering the scarcity of research on protein‐related claims despite an increase in demand, production, and consumption of protein‐enriched foods in the last decades (Wilson, 2019).

## 2 STUDY 1 (BEHAVIORAL STUDY)

### 2.1 Material and methods

#### 2.1.1 Participants

Participation in the study was voluntary, and participants were paid a €10 flat fee for their participation. For this study, we invited 113 participants of which three were excluded due to technical problems. Participants were invited via the hroot database (Bock et al., 2014) of the BonnEconLab. Registration in this database is voluntary and open to anyone. The final sample consisted of 110 participants (*M*_age_ = 23.66, *SD*_age_ = 3.25 years old; 67 women). Participants were asked to get around 6−8 h sleep the night before the experiment (indicated sleep hours *M* = 7.56, *SD* = 0.78 h) and to not eat 3 h before the experiment (indicated hours before the last meal *M* = 5.12, *SD* = 3.71 h; perceived hunger level on a 10‐point scale *M* = 5.42, *SD* = 2.31). We recruited only participants who liked milk‐mix drinks, had no neurological/psychiatric/psychological/metabolic conditions, no current upper‐respiratory infection, no food allergies, no intolerances, no conditions known to affect metabolism, and with a Body Mass Index (BMI) between 17.5 and 30 kg/m^2^ (*M*_BMI_ = 24.08, *SD*_BMI_ = 2.39 kg/m^2^, calculated by self‐reported weight and height).

#### 2.1.2 Study design

Data collection took place at the BonnEconLab at the University of Bonn, Germany. Upon arrival in the lab, participants were randomly assigned to either the fat‐claim or protein‐claim conditions. In total, 57 participants were assigned to the protein‐claim condition and 53 were assigned to the fat‐claim condition. The experiment comprised four main parts. In the first part, participants completed a survey containing questions assessing task comprehension, sociodemographic questions, and questions assessing baseline levels of hunger (on a 10‐point scale scale; 1 = not at all, 10 = very much), hours of sleep on the night before the experiment, and emotional valence and arousal (using the corresponding Self‐Assessment Manikin subscales from Bradley and Lang, 1994). There were no significant differences between groups in terms of age, BMI, baseline hunger, indicated hours of sleep, and emotional arousal and valence. There were also similar number of men and women assigned to each condition (see Supplementary Table S1).

Next, they were given information^2^ about the meaning of “fat‐reduced”/“protein‐rich” claims and were asked to rate their expectations regarding a conventional milk‐mix drink and a milk‐mix drink with a “fat‐reduced”/“protein‐rich” claim depending on the assigned condition. Participants rated expectations in terms of healthiness, taste pleasantness, satiating quality, needed amount of consumption to feel full, and wanting on a 9‐point scale (1 = not at all, 9 = very much).

**FIGURE 1:**
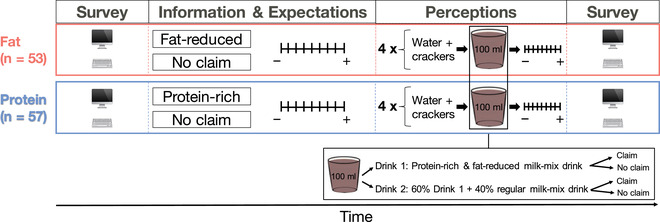
Behavioral study design. Each participant rated expected and perceived attributes about a milk‐mix drink with and without a nutrition claim on a 9‐point scale. Claims were “fat‐reduced” (red, *n* = 53 participants) or “protein‐rich” (blue, *n* = 57 participants). Each participant sampled two drinks, each presented once with and once without a nutrition claim. Drink order was fixed: Drink 1 was presented in the first and fourth round, and Drink 2 in the second and third round. Order of nutrition claim presence was counterbalanced across participants.

In the third part, participants sampled two different drinks, once presented with a nutrition claim (“fat‐reduced milk‐mix drink”/“protein‐rich milk‐mix drink”) and once without any nutrition claim (“milk‐mix drink”). In both conditions participants sampled the same drinks so that any observed difference in ratings between conditions could be attributed to the type of the claim. The drinks used in the study were chocolate‐flavored milk‐mix drinks found in the German market. One drink was a protein‐rich and fat‐reduced chocolate milk drink from Arla (Drink 1), and the other was a mixture of Drink 1 and a conventional chocolate milk drink from Müller (Drink 2). To avoid deception of participants, we prepared Drink 2 as a mixture such that it could be claimed to be “protein‐rich” and “fat‐reduced” (European Union Parliament, 2006). Participants were instructed how to swirl each drink in their mouth for ~10 s and to concentrate on the taste of the drinks. Sampling was done in four rounds; in each round, 100 ml of one drink was presented to the participants ad libitum. Drink 1 was presented in the first and fourth round, whereas Drink 2 was presented in the second and third round; the order of the nutrition claim presentation was counterbalanced across participants. Before sampling each drink, participants ate saltine crackers and drank some still water to cleanse their palate and reduce taste spillover effects between trials. After each sampling, participants rated the perceived taste pleasantness, healthiness, satiating quality, needed amount of consumption to feel full, and wanting; all ratings were assessed on a 9‐point scale (1 = not at all, 9 = very much; see Figure 1 for a representation of the design).

In the fourth part, participants completed a survey with questions about their general attitude toward food with nutrition claims and nutrition labels/claims and indicated which of the sampled drinks they preferred.

### 2.2 Statistical analyses

All behavioral data analyses were performed with R programming language (R Core Team, 2020) and RStudio version 4.0.3 (RStudio Team, 2019) using *lme4* (Bates et al., 2015)*, nlme* (Pinheiro et al., 2020)*, ggplot2* (Wickham, 2016)*, GGally* (Schloerke et al., 2021)*, lsmeans* (Lenth, 2016)*, reshape2* (Wickham, 2007)*, readxl* (Wickham & Bryan, 2019)*, sjPlot* (Lüdecke, 2021)*, dplyr* (Wickham et al., 2021), and *TOSTER* (Lakens, 2017).

First, to assess the effect of the presence (Yes/No) and type (Protein/Fat) of the nutrition claim, we estimated mixed‐effects regression models with expectation and perception ratings as dependent variables, nutrition claim presence (1 = Yes, 0 = No), nutrition claim type (1 = Protein, 0 = Fat), their interaction, and drink type (1 = Drink 2, −1 = Drink 1; to assess claim effects across drinks) as explanatory variables. In all models, we added an intercept per participant to control for interindividual differences in average ratings. To supplement our findings, for null results we also conducted equivalence tests by using the Two One‐Sided Test (TOST) procedure implemented in the *TOSTER* package in R (Lakens, 2017).

Second, we explored the association between claim effects and gender. To this end, we performed linear regression analyses where we included Gender (1 = Man, 0 = Woman) and Condition (1 = Protein, 0 = Fat) as explanatory variables, and claim effects as the dependent variable. We calculated claim effects for every attribute of interest by subtracting the ratings for the drinks without claims from the ratings for drinks with claims *(X_claim_ – X_no claim_)*. Regression analyses were performed separately for each attribute.

Third, we assessed whether the presence and type of the nutrition claim have an effect on the overall preference for the drinks. To this end, we compared the percentage of participants that preferred the drinks presented with a nutrition claim with the percentage of participants that preferred the drinks presented without a nutrition claim using a binomial test.

Fourth, we calculated claim prediction errors, that is, the differences in perceived and expected claim effect regarding all the assessed qualities as follows:

$$\begin{array}{ccc} & & \left[Perceived\;X\;claim-Perceived\;X\;no\;claim\right]\\ & & \;-\;\left[Expected\;X\;claim-Expected\;X\;no\;claim\right], \end{array}$$

where *X* is substituted with the ratings for the assessed qualities, namely taste pleasantness, healthiness, and satiating quality. We tested these prediction errors against zero using one‐sample *t*‐tests.

Finally, we assessed whether differences in expectation, perception ratings, or prediction errors could explain preference for drinks with a nutrition claim (assessed post‐sampling). To this end, we estimated a logistic model where we included preference for the drink with the claim (1 = Yes, 0 = No) as the dependent variable, and the type of claim (Protein = 1, Fat = 0), difference in expected and perceived taste pleasantness, healthiness, and satiating quality, as well as differences in prediction errors as explanatory variables. Differences were calculated by subtracting the ratings for the drink presented without a nutrition claim from the ratings for the drinks presented with a nutrition claim (*X*_claim_ – *X*_no claim_, where *X* is substituted for the average ratings for the respective attribute for each participant).

### 2.3 Results

#### 2.3.1 Effect of nutrition claims on expected and perceived food attributes

**FIGURE 2:**
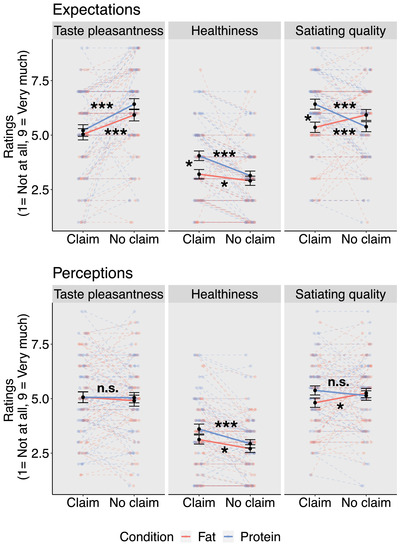
Effect of nutrition claims on expected (upper panel) and perceived (lower panel) attribute ratings. Black dots indicate means across participants, red and blue dots are mean ratings per participant. Error bars represent the standard error of the mean. Tukey's tests were used for pairwise comparisons. *n*_Protein_ = 57, *n*_Fat_ = 53; ***p < .001, **p < .01, *p < .05, n.s.: not significant.

Mixed‐effects regressions revealed that nutrition claims decreased taste pleasantness expectations (*χ* ^2^_claim (1)_ = 33.45, *p* < .001), increased healthiness expectations (*χ* ^2^_claim (1)_ = 7.86, *p* = .005) and more so in the protein‐claim condition (*χ* ^2^_claim x type of claim (1)_ = 17.001, *p* < .001), and changed expected satiating quality ratings depending on the type of the claim (*χ* ^2^_claim (1)_ = 28.61, *p* < .001; *χ* ^2^_claim x type of claim (1)_ = 119.04, *p* < .001; see Figure 2 and Table 1). Nutrition claims did not have an effect on perceived taste pleasantness (*χ* ^2^_claim (1)_ = 0.51, *p* = .47) but significantly increased perceived healthiness (*χ* ^2^_claim (1)_ = 9.05, *p* = .003). Nutrition claims influenced perceived satiating quality ratings, but differently depending on the type of the claim (*χ* ^2^_claim (1)_ = 6.98, *p* = .008; *χ* ^2^_claim x type of claim (1)_ = 8.68, *p* = .003) (see Figure 2 and Table 1).

**TABLE 1:** Effect of nutrition claims on expected and perceived food attributes

	DV: Expected taste pleasantness			DV: Expected healthiness			DV: Expected satiating quality		
Fixed effects	*B* (*SE*)	95% CI	*p*	*B* (*SE*)	95% CI	*p*	*B* (*SE*)	95% CI	*p*
Intercept	5.92 (*0.24*)	[5.45, 6.40]	< .001	2.91 (*0.20*)	[2.51, 3.30]	<.001	5.92 (*0.23*)	[5.47, 6.37]	<.001
Claim (1 = Yes, 0 = No)	−0.89 (*0.15*)	[−1.19, −0.59]	< .001	0.30 (*0.11*)	[0.09, 0.51]	.005	−0.57 (*0.11*)	[−0.77, −0.36]	<.001
Condition (1 = Protein, 0 = Fat)	0.49 (*0.34*)	[−0.17, 1.16]	.148	0.23 (*0.28*)	[−0.33, 0.78]	.421	−0.55 (*0.32*)	[−1.17, 0.08]	.087
Claim × Condition	−0.32 (*0.21*)	[−0.74, 0.10]	.134	0.62 (*0.15*)	[0.32, 0.91]	<.001	1.61 (*0.15*)	[1.32, 1.89]	<.001
**Random effects**	**σ^2^**	**τ_00_**	**ICC**	**σ^2^**	**τ_00_**	**ICC**	**σ^2^**	**τ_00_**	**ICC**
Intercept (ID)	1.25	2.52	.67	0.61	1.87	.75	0.59	2.48	.81
**Model**									
Marginal *R* ^2^/Conditional *R* ^2^	.076/.695			.072/.771			.060/.819		

	**DV: Perceived taste pleasantness**			**DV: Perceived healthiness**			**DV: Perceived satiating quality**		
**Fixed effects**	***B* (*SE*)**	**95% CI**	***p***	***B* (*SE*)**	**95% CI**	***p***	***B* (*SE*)**	**95% CI**	***p***
Intercept	4.91 (*0.26*)	[4.40, 5.41]	< .001	2.71 (*0.20*)	[2.31, 3.11]	<.001	5.25 (*0.21*)	[4.83, 5.68]	<.001
Claim (1 = Yes, 0 = No)	0.16 (*0.22*)	[−0.28, 0.60]	.475	0.42 (*0.14*)	[0.14, 0.69]	.003	−0.44 (*0.17*)	[−0.77, −0.11]	.009
Condition (1 = Protein, 0 = Fat)	0.13 (*0.36*)	[−0.57, 0.83]	.713	0.21 (*0.28*)	[−0.34, 0.77]	.452	−0.12 (*0.30*)	[−0.71, 0.47]	.690
Claim × Condition	−0.13 (*0.31*)	[−0.74, 0.48]	.678	0.27 (*0.1*9)	[−0.11, 0.65]	.160	0.69 (*0.23*)	[0.23, 1.15]	.003
Drink (1 = Drink 2, −1 = Drink 1)	0.29 (*0.08*)	[0.14, 0.44]	< .001	−0.06 (*0.05*)	[−0.15, 0.03]	.212	0.23 (*0.06*)	[0.11, 0.34]	<.001
**Random effects**	**σ^2^**	**τ_00_**	**ICC**	**σ^2^**	**τ_00_**	**ICC**	**σ^2^**	**τ_00_**	**ICC**
Intercept (ID)	2.66	2.17	.45	1.01	1.71	.63	1.49	1.70	.53
**Model**									
Marginal *R* ^2^/Conditional *R* ^2^	.018/.459			.041/.645			.029/.546		

*Notes*: Effects are estimated using mixed effects regression models. *p‐v*alues are calculated based on the *t*‐statistics using the normal distribution function. τ_00_ denotes the variance in intercepts, σ^2^ denotes the residual variance. *n*_Protein_ = 57, *n*_Fat_ = 53.

ID: participant ID; DV: dependent variable; *B*: unstandardized estimate; *SE*: standard error of the estimate; CI: confidence interval; ICC: intraclass correlation coefficient.

Equivalence testing revealed that the difference in perceived taste pleasantness ratings for drinks presented with and without a nutrition claim is statistically equivalent to zero given equivalence bounds of Cohen's *d* = ± 0.23, at 5% alpha level (*t*_(109)_ = −1.77, 90% CI [−0.137, 0.309], *p* = .04; data pooled across claims).

Linear regression analyses revealed an association between gender and claim effects on expected satiating quality (*F*_Gender x Condition (1)_ = 4.24, *p* = .04). More specifically, the difference in claim effects between the protein and the fat condition was significantly higher in women than in men (*B*_Gender x Claim_ = −1.08, *SE* = 0.52, 95% CI [−2.11, −0.04], *p* = .04). Further pairwise comparisons also indicated that the effect of the “protein‐rich” claim on expected satiation was significantly higher in women than in men (Tukey‐adjusted comparison: *t*_(106)_ *=* 2.83, *p* = .03). There were no associations between gender and other expectation and perception ratings (see Supplementary Table S2 and Supplementary Figure S1 for full model results).

#### 2.3.2 Prediction errors (perception *vs*. expectation)

As effects of the claims on expectations and perception differed, we subtracted expectation ratings from perception ratings (*i.e*., prediction errors) and tested the difference against zero (see Section 2.2). Prediction errors for taste pleasantness were positive for both claims (“protein‐rich” claim taste pleasantness prediction error: *t*_(56)_ = 3.86, *p* = .0003; “fat‐reduced” claim taste pleasantness prediction error *t*_(52)_ = 3.69, *p* = .0005) and did not significantly differ between them (group comparison *t*_(108)_ = −0.42, *p* = .674). In other words, participants expected drinks with the claim to taste worse than they actually did. Prediction errors for healthiness were not significant for either claim (“protein‐rich” claim *t*_(56)_ = −1.46, *p* = .149; “fat‐reduced” claim *t*_(52)_ = 0.48, *p* = .633), whereas prediction errors for satiating quality were only significant for the “protein‐rich” claim (*t*_(56)_ = −3.61, *p* = .001; “fat‐reduced” claim: *t*_(52)_ = 0.42, *p* = .679; see Supplementary Figure S2).

#### 2.3.3 Preference for drinks

55.45% of participants preferred a drink with a claim (independent of the type) and 38.18% preferred a drink without a claim (6.36% indicated no preference). This was not different from chance level (proportion preferring drink with claim = 0.59, 95% CI [0.49, 0.69], *p* = .076; binomial test). Preference for drinks with a claim was explained by the perceived pleasantness difference (*OR* = 3.30, *SE* = 0.87, 95% CI [2.07, 5.87], *p* < .001) and pleasantness prediction errors (*OR* = 1.30, *SE* = 0.14, 95% CI [1.07, 1.62], *p* = .012), but not by expected pleasantness differences (*OR* = 1.09, *SE* = 0.11, 95% CI [0.89, 1.34], *p* = .410; see Supplementary Table S3).

## 3 STUDY 2 (FMRI STUDY)

### 3.1 Material and methods

#### 3.1.1 Participants

Participation in the study was voluntary, and participants were paid a fee of €15 per hour for their participation. Additionally, they received one of the milk‐mix drinks they encountered in the experiment. The participants for this study were recruited via e‐mail from the participant pool of the Life and Brain research center (a database where anyone can sign up) and flyers posted online on social media. The exclusion criteria were: not liking milk‐mix drinks, being underweight or having obesity (BMI below 18 or above 30 kg/m^2^), standard MRI exclusion criteria (metal/medical implants, claustrophobia), having neurological/psychiatric/psychological or being on medication for neurological/psychiatric/psychological/metabolic conditions, having a current upper‐respiratory infection, and having food allergies, intolerances, diabetes or any condition known to affect taste perception and/or metabolism. In total, 42 participants participated in Study 2. From those, three were excluded from the behavioral data analyses and another six (nine in total) from the fMRI data analyses. Reasons for exclusions were: incomplete experiment, excessive movement (>3 mm), anatomical alterations discovered during data analyses, and technical problems during the experiment. The final sample of Study 2 consisted of 39 participants (*M*_age_ = 26.41, *SD*_age_ = 10.68 years old; *M*_BMI_ = 23.41, *SD*_BMI_ = 2.83 kg/m^2^, calculated by assessed weight and height; 19 women) for the behavioral and 33 participants (14 women) for the fMRI data analyses. A sensitivity power analysis performed using the G*Power software (Faul et al., 2009) revealed that this sample size would allow us to detect an effect size of *d*z ≥ 0.503 with *α* = 5% and 1 – β = 0.8 (80% power), in a two‐tailed paired *t*‐test.

Participants were asked to get a good night's sleep (approximately 6−8 h) before the experiment day (indicated sleep hours *M* = 7.27, *SD* = 1.31 h) and were asked to eat no later than 2 h before the experiment, so that they would be somewhat hungry during the experiment (indicated hours before the last meal *M* = 2.72, *SD* = 1.26 h; perceived hunger level on an 11‐point scale *M* = 5.97, *SD* = 1.33).

#### 3.1.2 Study design

Data collection took place at the Life and Brain Research Center in Bonn, Germany. The study consisted of four parts. Like in Study 1, the first part of Study 2 consisted of a survey that included questions assessing task comprehension, sociodemographic questions, and questions assessing baseline levels of hunger (on an 11‐point scale; 1 = not at all, 11 = very much), perceived stress (on a 9‐point scale; 0 = not at all, 9 = very much), hours of sleep on the night before the experiment, emotional valence and arousal (using the corresponding Self‐Assessment Manikin subscales from Bradley & Lang, 1994).

Since in Study 1 we did not find a difference in expected and perceived pleasantness between the “fat‐reduced” and “protein‐rich” claims, in Study 2 we did not compare the neural effects on pleasantness and valuation of both claims. Instead, in this study we only used the “protein‐rich” claim. Like in Study 1, we gave participants information about the meaning of the claim and asked them to indicate their expectations about the taste pleasantness, healthiness, and satiating quality of protein‐rich and conventional milk‐mix drinks (on a 9‐point scale; same scale as in Study 1). Considering that in Study 1 we found that nutrition claims affect some attributes and not others, in Study 2 we additionally assessed their effect on valuation. To this end, we asked participants to indicate their hypothetical willingness to pay (WTP) for protein‐rich and conventional milk‐mix drinks; they could indicate any amount ranging from 0 to €3 (€3 is approximately 30% more than the retail price for milk‐mix drinks).

**FIGURE 3:**
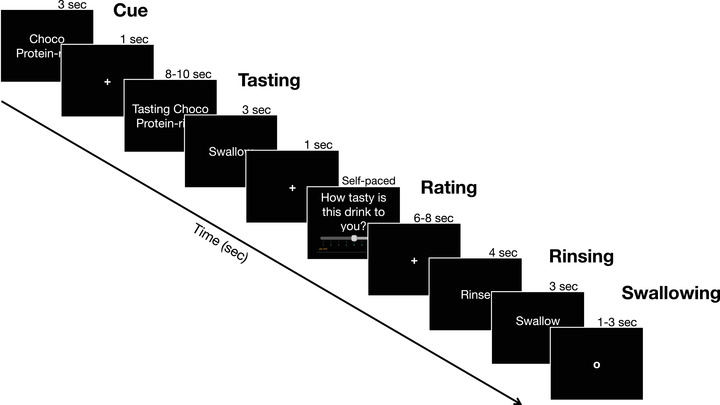
Timeline of a trial in the fMRI task. Each participant completed 48 trials in total, 12 for each *flavor+ claim presence* combination (12 claim chocolate, 12 no claim chocolate, 12 claim vanilla, 12 no claim vanilla). The flavor of the drinks as well as the order of the claim presentation was randomized for each participant with the restriction that the same combination of *flavor + claim* presence could not be presented two times in a row. Taste pleasantness ratings for each trial were self‐paced and participants were told to rate the drinks as fast as they could.

The second part of Study 2 was the fMRI experiment, which consisted of a taste‐rating task, whereby participants were delivered different drinks while lying inside the MRI scanner and were asked to taste and rate the pleasantness of each delivered drink. The drinks were delivered using an in‐house‐built electronic syringe pump system used in a previous study by Schmidt et al. (2017). Participants were delivered two protein‐rich drinks from Arla: one with chocolate (same as in Study 1) and one with vanilla flavor. Each drink was presented 12 times with and without the “protein‐rich” claim (see Figure 3). In total, participants completed 48 trials (2 flavors × 2 conditions × 12 repetitions). The flavor of the drinks as well as the order of the claim presentation was randomized for each participant with the restriction that the same combination of *flavor + claim* presence could not be presented two times in a row.

Each trial started with a cue representation that indicated which drink was going to be delivered. The trial continued with the delivery of 1 ml (delivered in 2.67 s) of the cued drink and a tasting period where participants were asked to concentrate on the taste of the drink. Next, participants were instructed to swallow the drink and rate its pleasantness (on a 9‐point scale, like in Study 1). Participants were told to rate the drinks as fast as possible so that their ratings reflect the momentarily perceived pleasantness (across participants average rating per trial ranged from Min = 1.65 s to Max = 5.33 s; *M*_Rating_ = 3.15 s, Median_Rating_ = 2.84 s, *SD* = 0.94 s). At the end of the trial, participants were delivered a tasteless rinsing solution (for details on the rinsing solution preparation see Supplementary Material Section 2.1.1), which they were then instructed to swallow (see Figure 3); participants were reminded to strictly follow the instructions presented on the screen, and only swallow when told to do so. The rinsing solution was used to avoid spillover over trials, and to provide a baseline condition later used to assess taste responses at the neural level. To make sure that participants understood the task and were comfortable with it, we ran a few test trials prior to starting scanning.

The third part, similar to the first part, was completed outside the scanner and consisted of another survey. This post‐fMRI survey contained questions concerning the drinks that participants sampled during the fMRI task and their attitudes toward labels and claims (like in Study 1). Moreover, in this part, participants were asked to complete the Dutch Eating Behaviour Questionnaire (DEBQ; Grunert, 1989; van Strien et al., 1986), Food Neophobia Scale (FNS; Pliner & Hobden, 1992), and the Brief Self‐Control Scale (BSCS; Bertrams & Dickhäuser, 2009; Tangney et al., 2004). In this study, we only report descriptive statistics for the DEBQ questionnaire, as the other questionnaires were collected for a different project. DEBQ was included only for better characterization of the eating styles of our sample.

The fourth and final part was a sweet taste sensitivity test, used to assess participants’ ability to taste sweetness. For this, we estimated sucrose recognition thresholds using an adaptive procedure based on QUEST+ (Watson, 2017), which is an extension of an established protocol using a yes–no task (Höchenberger & Ohla, 2017, 2019) (for details on the procedure, see Supplementary Material Section 2.1.2). This test was also included only for better sample characterization.

#### 3.1.3 fMRI data acquisition

The MRI data were acquired on a 3T Siemens Trio scanner with a 32‐channel head coil. Participants were shown the fMRI task via a mirror that was mounted on the head coil and adjusted so that the participants could correctly see the screen positioned behind their heads. Responses were indicated using controllers in both hands. The scanning sequence consisted of a gradient field map (GFM), a functional scan, and T1‐weighted structural images at the end. The GFM sequence was acquired using a double echo sequence with the first echo time (TE) = 4.92 ms and second TE = 7.38 ms, repetition time (TR) = 392 ms, field of view (FoV) = 92 mm, and flip angle = 60°. Functional images were acquired using an echoplanar imaging (EPI) sequence with the following parameters: TR = 2500 ms, TE = 30 ms, flip angle = 90°, FoV = 192 mm, voxel size (*x*, *y*, *z*) = 2 mm × 2 mm × 3 mm^3^, number of slices = 37. The slices were acquired on an axial orientation in an ascending order. The number of acquired images differed across participants (as certain stages of the fMRI task were self‐paced), ranging from 678 to 763 images with *M* = 712.71 images. Structural images were acquired with the following parameters: TR = 1660 ms, TE = 2.54 ms, flip angle = 9°, voxel size (*x*, *y*, *z*) = 0.8 mm × 0.8 mm × 0.8 mm, FoV = 256 mm. The images were acquired on a sagittal orientation and a total of 208 images were acquired per participant.

#### 3.1.4 fMRI data preprocessing

All MRI data preprocessing was conducted using the SPM12 software package (Wellcome Department of Imaging Neuroscience, Institute of Neurology, London, UK) based on MATLAB R2020b. Preprocessing was done as follows: First, the images were slice‐time corrected with the first image as the reference. Second, the data were corrected for motion. The realignment parameters were visually inspected, and all the participants that at any point during the session moved more than the voxel size (>3 mm) from their initial position (first functional scan) were excluded. Next, the images were unwarped using the GFMs acquired prior to the functional scans, coregistered to the individual high‐resolution T1‐weighted structural images, transformed into the Montreal Neurological Institute (MNI) template space, and resampled to 3 × 3 × 3 mm^3^ voxel size. To account for interindividual anatomical differences and reduce the thermal noise, the images were smoothed with a Gaussian kernel with full width at half maximum (FWHM) of 8 mm. To filter out the low‐frequency noise, a high‐pass temporal filter of 128 s was used. The quality of the functional and structural data was checked using the Check Reg function in SPM12, the SPM CAT12 toolbox (r1184, http://www.neuro.uni‐jena.de/cat/), and the MRI Quality Control (MRIqc) tool to extract objective quality metrics (Esteban et al., 2017).

### 3.2 Statistical analyses

Consistent with Study 1, behavioral data analyses were performed using R and RStudio version 4.0.3 (RStudio Team, 2019). fMRI data were analyzed using SPM12 and SPM8 (Wellcome Department of Imaging Neuroscience, Institute of Neurology, London, UK) under MATLAB R2020b. We used MarsBar (Brett et al., 2002), AAL3 (Rolls et al., 2020), Anatomy (Eickhoff et al., 2005), WFU Pickatlas (RRID:SCR_007378; https://www.nitrc.org/projects/wfu_pickatlas/) (Maldjian et al., 2003), and gPPI (McLaren et al., 2012) SPM toolboxes.

#### 3.2.1 Behavioral analyses

Like in Study 1, we first assessed the effect of the “protein‐rich” claim on expectations and perceived taste pleasantness. To this end, we estimated separate mixed‐effects linear regression models with the respective ratings as the dependent variable, claim presence (1 = Yes, 0 = No) as the explanatory variable, and a random intercept term per participant to account for interindividual differences in average ratings. In the model assessing the effect of claim on perceived pleasantness, we additionally included flavor (1 = Chocolate, −1 = Vanilla; to assess claim effects across flavors), and trial number as explanatory variables. Like in Study 1, to supplement our findings, for null results we also conducted equivalence tests using the TOST procedure.

Second, we assessed the effect of the “protein‐rich” claim on subjective value (as assessed via the WTP measure). To this end, we estimated a mixed‐effects regression model with WTP ratings as the dependent variable, claim presence (1 = Yes, 0 = No) as the explanatory variable, and a random intercept term per participant to account for interindividual differences in average WTP. To assess which attributes related to valuation, and whether this changed depending on the claim, we estimated linear regression models with mean WTP ratings as dependent variable and mean expectation ratings as explanatory variables.

Third, like in Study 1, we explored the association between gender and claim effects on each attribute of interest (expectations, perceived taste pleasantness, WTP) using linear regression analyses. In these analyses we included Gender (1 = Man, 0 = Woman) as the explanatory variable, and claim effects as the dependent variable.

Fourth, we assessed whether claim effects on expectations explained claim effects on perceived taste pleasantness. To this end, we estimated a linear regression model with average perceived differences in taste pleasantness as the dependent variable, and the average expected differences in taste pleasantness, healthiness, and expected satiating quality as explanatory variables.

Fifth, similarly to Study 1, we calculated taste pleasantness prediction errors, and assessed whether they are different from zero using a one‐sample *t*‐test.

Finally, as in Study 1, we assessed the effect of claim on preference for drinks (assessed post‐fMRI). To this end, we counted and compared the frequency of preferring a drink with the claim and preferring a drink without the claim, using a binomial test. Furthermore, we assessed whether preference for drinks with a claim could be explained by expectations and perceived taste pleasantness ratings. To this end, we estimated a logistic regression with preference for a drink with the “protein‐rich” claim (1 = Yes, 0 = No) as the dependent variable, and the differences in expectations (taste pleasantness, healthiness, satiating quality), perceived taste pleasantness, and taste pleasantness prediction errors as explanatory variables.

#### 3.2.2 fMRI analyses

##### Analysis strategy

We used the following strategy. First, we checked whether our paradigm evoked the expected responses in brain regions associated with taste and flavor processing. Second, we tested whether the “protein‐rich” claim modulated activity in brain regions associated with valuation. These regions were identified based on previous work (vS/Nucleus accumbens [NAcc], vmPFC, dlPFC, lateral OFC). Finally, we assessed the impact of the claim on functional connectivity. To assess effects of interest on the above‐mentioned ROIs, we performed small‐volume‐correction (SVC) analyses. We consider activations as significant if they survive *p* < .05 with family‐wise (FWE) correction for multiple comparisons; for whole‐brain analyses, this correction was applied at the cluster level, based on a threshold of *p* = .001 uncorrected at the voxel level (cluster‐forming threshold); for SVC analyses, this correction was applied at the peak level.

##### GLM definition and contrasts of interest

To assess the effect of claims on neural activity during tasting and swallowing of the drinks, we estimated a GLM including regressors for: cue claim, cue no claim, tasting drinks presented with a claim, tasting drinks presented without a claim, swallowing drinks presented with a claim, swallowing drinks presented without a claim, rinsing, swallowing rinsing solution, rating, and movement (three for translation, three for rotation). Every regressor modeled responses from event onset until event offset. We estimated this model for every participant and for each we calculated eight contrasts: tasting drinks *vs*. rinsing, swallowing drinks *vs*. swallowing rinsing solution, viewing cue for drinks with *vs*. without the claim and vice versa, tasting drinks with *vs*. without the claim and vice versa, and swallowing drinks with *vs*. without the claim and vice versa. These calculated contrasts were subjected to one‐sample *t*‐tests (2nd‐level analyses).

##### Assessing taste and flavor response in brain regions of interest (ROIs)

Several regions including the OFC, insula, frontal and rolandic operculum, ACC, amygdala, caudate, putamen, pallidum, and thalamus have been associated with taste and flavor processing (Lundström et al., 2011; Veldhuizen et al., 2011). To assess whether our task evoked taste and flavor responses in these regions, we applied SVC to the whole‐brain contrasts tasting *vs*. rinsing and swallowing drinks *vs*. swallowing rinsing solution. To restrict the number of independent tests and thus reduce the probability of type I errors, we constructed a common mask of these anatomical ROIs using the WFU Pickatlas tool (Maldjian et al., 2003) and applied SVC over the mask volume (see Supplementary Figure S3a); this approach has also been used in previous studies (e.g., van Rijn et al., 2018).

##### Assessing the effect of the “protein‐rich” claim in brain ROIs

We applied SVC to the whole‐brain results of our GLM to restrict analyses in a priori defined regions associated with valuation, including bilateral vS/NAcc ([*x*, *y*, *z*] = [−12, 10, −2], and [*x*, *y*, *z*] = [12, 10, −2], both 10 mm as in Knutson et al., 2008; Linder et al., 2010), vmPFC ([*x*, *y*, *z*] = [2, 46, −8], 10 mm as in Bartra et al., 2013), left dlPFC (Hare et al., 2009, 2011; [*x*, *y*, *z*] = [−48, 15, 24], 10 mm as in Enax et al., 2015a), and left lateral OFC ([*x*, *y*, *z*] = [−22, 34, −8], 10 mm as in Kringelbach et al., 2003). As for taste and flavor ROIs, to restrict the number of independent tests, we constructed a common mask of these valuation‐associated ROIs (see Supplementary Figure S3b) and applied SVC over the mask volume. All ROIs were built in MarsBar as spheres and were combined using the ImCalc function in SPM.

##### Assessing the effect of the “protein‐rich” claim on functional connectivity

To test if the “protein‐rich” claim impacts functional connectivity patterns in the brain, we conducted Psycho Physiological Interaction (PPI) analyses using the *gPPI* toolbox (McLaren et al., 2012). In these analyses, we used regions that were more active in response to claim *vs*. no claim (and vice versa) as seed regions and searched for functional connectivity changes across the whole brain. For details on the PPI analyses, see Supplementary Material (Section 2.1.3).

### 3.3 Results

#### 3.3.1 Behavioral results

##### Eating behavior and sweet taste sensitivity

The acquired baseline ratings, the DEBQ questionnaire subscores, and the sweet taste thresholds are summarized in Supplementary Table S4. Mean DEBQ subscores fell within ±3 SD of the published norms for the German population (Nagl et al., 2016), and therefore indicated that our sample exhibited an eating behavior within the norm. Sweet taste thresholds were numerically lower, indicating a higher sensitivity than in previous reports that used a similar procedure based on QUEST (Hardikar et al., 2017; Höchenberger & Ohla, 2017, 2019). Nevertheless, the results indicated that participants were able to recognize sweet taste (for a discussion on the taste test results, see Supplementary Material Section 2.2.2).

##### Effect of nutrition claim on expectations, perceived taste pleasantness, and valuation

**FIGURE 4:**
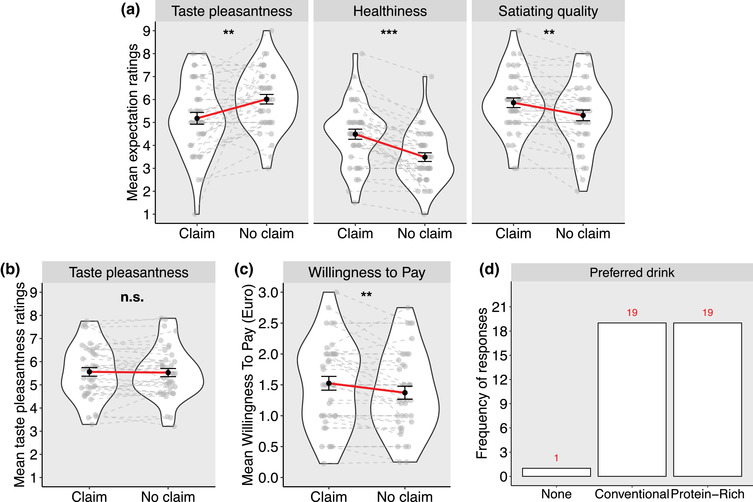
Effect of the “protein‐rich” nutrition claim on expectations (a), perceived pleasantness (b), willingness to pay (c), and overall preference (d). Black dots are mean values across participants, whereas gray dots are individual mean values. The red line connects the mean ratings in both conditions, whereas dashed gray lines connect the mean ratings of each participant. Ratings and frequencies are pooled across both flavors (chocolate, vanilla). Error bars represent the standard error of the mean . *n* = 39; ***p < .001; **p < .01; n.s.: not significant.

Our mixed effects linear models revealed that, consistent with Study 1 results, participants expected a protein‐rich drink to be less tasty (*χ* ^2^_(1)_= 11.94, *p* = .0005; *B*_claim_ = −0.83, *SE* = 0.24, 95% CI [−1.31, −0.35], *p* = .001), more healthy (*χ* ^2^_(1)_= 41.75, *p* < .001; *B*_claim_ = 1.00, *SE* = 0.15, 95% CI [0.69, 1.31], *p* < .001), and more satiating than a conventional drink (*χ* ^2^_(1)_= 11.149, *p* = .001; *B*_Claim_ = 0.55, *SE* = 0.17, 95% CI [0.22, 0.88], *p* = .001) (Figure 4a).

Participants rated the perceived pleasantness of the drink similarly when it was presented with and without the “protein‐rich” claim (*χ* ^2^_(1)_ = 0.225, *p* = .636; see Figure 4b), corroborating the findings from Study 1 (see Table 2). Equivalence testing revealed that the difference in perceived taste pleasantness ratings for drinks presented with and without the “protein‐rich” claim is statistically equivalent to zero given equivalence bounds of Cohen's *d* =  ± 0.34 and alpha of 5% (*t*_(38)_ = 1.746, 90% CI [−0.175, 0.111], *p* = .044).

**TABLE 2:** Effects of a “protein‐rich” nutrition claim on perceived taste pleasantness ratings

	DV: Perceived taste pleasantness					
Fixed effects	*B* (*SE*)	95% CI	*p*	*B* (*SE*)	95% CI	*p*
Intercept	5.65 (*0.19*)	[5.28, 6.01]	<.001	5.65 (*0.19*)	[5.28, 6.01]	<.001
Claim (1 = Yes, 0 = No)	−0.03 (*0.06*)	[−0.16, 0.09]	.636	−0.03 (*0.06*)	[−0.16, 0.09]	.636
Trial number	−0.004 (*0.002*)	[−0.01, 0.001]	.127	−0.003 (*0.002*)	[−0.01, 0.001]	.130
Flavor (1 = Chocolate, −1 = Vanilla)	0.30 (*0.03*)	[0.24, 0.36]	<.001	0.29 (*0.05*)	[0.20, 0.37]	<.001
Claim × Flavor				0.03 (*0.06*)	[−0.09, 0.16]	.591
**Random effects**						
	**σ^2^**	**τ_00_**	**ICC**	**σ^2^**	**τ_00_**	**ICC**
Intercept (ID)	1.90	1.16	.38	1.90	1.16	.38
**Model**						
Marginal *R* ^2^/Conditional *R* ^2^	.030/.399			.030/.399		

*Notes*: Effects are estimated using mixed‐effects linear regression models. *p*‐values are calculated based on the *t*‐statistics using the normal distribution function. τ_00_ denotes the variance in intercepts, σ^2^ denotes the residual variance; *n* = 39.

ID: participant ID; DV: dependent variable; *B*: unstandardized estimate; *SE*: standard error of the estimate; CI: confidence interval; ICC: intraclass correlation coefficient.

Participants were willing to pay significantly more for a protein‐rich drink than a conventional drink (*χ* ^2^_(1)_= 7.903, *p* = .005; *B*_claim_ = 0.15, *SE* = 0.05, 95% CI [0.05, 0.26], *p* = .006; see Figure 4c). Linear regression analyses indicated that for protein‐rich drinks, WTP was explained only by satiating quality ratings (*B* = 0.44, *SE* = 0.11, 95% CI [0.20, 0.67], *p* = .001), whereas for conventional drinks WTP was explained by expected taste pleasantness ratings (*B* = 0.21, *SE* = 0.10, 95% CI [0.004, 0.42], *p* = .046; see Supplementary Table S5).

Different than in Study 1, linear regression analyses revealed no significant associations between claim effects on attributes of interest (expectations, perceived pleasantness, WTP) and gender (see Supplementary Table S6).

##### Effect of expectations on perceived taste pleasantness

Linear regression results revealed that claim effects on expectations did not explain claim effects on perceived taste pleasantness (see Table 3).

**TABLE 3:** Relation between the “protein‐rich” claim effect on expectations with the claim effect on perceived taste pleasantness

	DV: Effect of claim on perceived taste pleasantness		
Fixed effects	*B* (*SE*)	95% CI	*p*
Intercept	−0.03 (*0.08*)	[−0.20, 0.14]	.707
Effect of claim on expected taste pleasantness	0.12 (*0.09*)	[−0.05, 0.30]	.158
Effect of claim on expected healthiness	0.09 (*0.09*)	[−0.10, 0.27]	.363
Effect of claim on expected satiating quality	−0.02 (*0.09*)	[−0.21, 0.17]	.843
**Model**			
*R* ^2^/adjusted *R* ^2^	.082/.003		

*Notes*: Effects are estimated using a linear regression model. Differences in expectation ratings are *z*‐scored; *n* = 39.

DV: dependent variable; *B*: unstandardized estimate; *SE*: standard error of the estimate; CI: confidence interval.

##### Prediction errors and preference for drinks

Consistent with Study 1, we found that taste pleasantness prediction errors were significantly larger than zero (*t*_(38)_ = 3.40, *p* = .002; see Supplementary Figure S4), suggesting that the “protein‐rich” claim reduced taste pleasantness less than expected.

We found that 48.72% of participants preferred a drink presented with the “protein‐rich” claim and 48.72% preferred a drink without a claim (2.56% indicated no preference; see Figure 4d). Different than in Study 1, our logistic regression analysis revealed that in Study 2, only perceived taste pleasantness difference explained preference for a drink with claim (*OR* = 3.04, *SE* = 1.71, 95% CI [1.21, 11.63], *p* = .048; see Supplementary Table S7 for all model results).

#### 3.3.2 fMRI results

##### Data quality check

Prior to analyzing the MRI data, we assessed its quality (see Section 3.1.4). Results are summarized in the Supplementary Material (Section 2.2.1, Supplementary Table X).

##### Taste and flavor response in the brain

Within regions associated with taste processing (see Section 3.2.2), tasting a drink *vs*. rinsing increased activation in bilateral caudate and left orbital gyrus, and swallowing drinks *vs*. swallowing rinsing solution increased activation in left insula, right operculum, and left anterior cingulate cortex (see Supplementary Table S8 and Supplementary Figure S5).

##### Effect of the “protein‐rich” claim on valuation and taste processing

We tested whether the “protein‐rich” claim modulated activity magnitude in brain regions previously associated with valuation and taste processing. Regions that exhibited an increased activity were used as seed regions in subsequent functional connectivity analyses.

**FIGURE 5:**
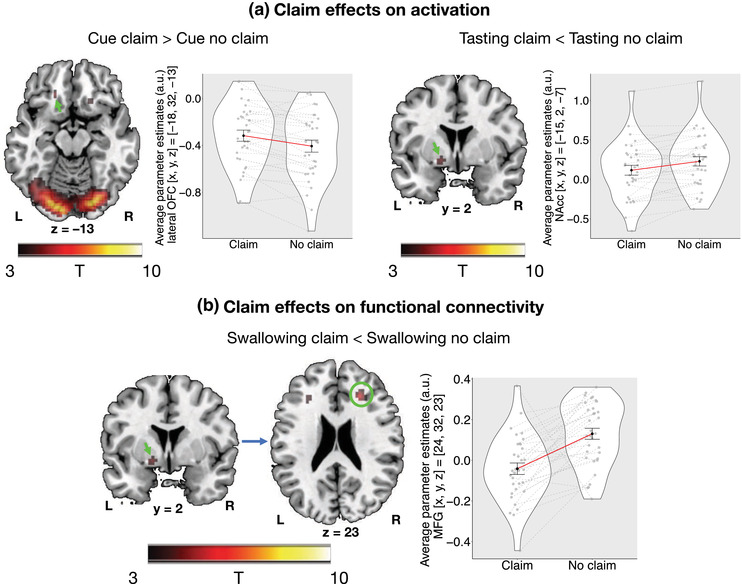
Claim effects on brain activation (a) and task‐dependent functional connectivity (b). (a) Viewing the “protein‐rich” cue claim *vs*. no claim was associated with an increased activity in a cluster in lateral OFC (green arrow upper left). Tasting drinks without *vs*. with the “protein‐rich” claim was associated with an increased activity in a cluster extending into the left NAcc (green arrow upper right). These activations survive thresholding at *p*_FWE_ < .05 when small volume correcting over regions associated with valuation (common valuation mask, see Methods). (b) Swallowing drinks without *vs*. with the “protein‐rich” claim was associated with an increased functional connectivity between the cluster extending into the left NAcc (green arrow) and a cluster in right middle frontal gyrus (green circle). This increased connectivity survives thresholding at *p*_FWE_ < .05 across the whole brain. T‐maps are overlayed on the ch2bet template. Black dots on the violin plots are mean parameter estimates across participants, whereas gray dots are individual mean parameter estimates. The red line connects the mean parameter estimates, whereas the dashed gray lines connect the individual mean parameter estimates. Error bars represent the standard error of the mean. L: left; R: right; a.u.: arbitrary units; *n* = 33.

*Claim > No claim, SVC analyses*: We found no significant activation difference in ROIs associated with valuation (vmPFC, bilateral vS/NAcc, left lateral OFC, dlPFC; see Section 3.2.2), neither for tasting nor for swallowing drinks presented with *vs*. without the “protein‐rich” claim. However, activation in a left lateral OFC cluster increased when viewing cue claim *vs*. no claim ([*x*, *y*, *z*] = [−18, 32, −13], *k* = 2, *z*‐value = 3.53, *T*‐value = 3.94, *p*_FWE_ < .05; see Figure 5a, left).

*Claim > no claim, functional connectivity*: There was no significantly increased functional connectivity between the lateral OFC cluster and the rest of the brain neither when viewing cues, tasting, or swallowing drinks.

*No claim > claim, SVC analyses*: We found no significant differences in the ROIs’ activity during the cue and swallow phases. On the other hand, during tasting, we found an increased activity in a cluster extending into the left NAcc and pallidum ([*x*, *y*, *z*] = [−15, 2, −7], *k* = 5, *z*‐value = 3.51, *T*‐value = 3.91, *p*_FWE_ < .05 for tasting drinks without *vs*. with the “protein‐rich” claim; see Figure 5a, right and Supplementary Figure S6 for additional coronal slices showing overlap with adjacent structures).

*No claim > claim, functional connectivity*: We found an increase in functional connectivity between the cluster extending into the left NAcc (see previous paragraph) and a cluster of the middle frontal gyrus when swallowing drinks without *vs*. with the “protein‐rich” claim ([*x*, *y*, *z*] = [24, 32, 23], *k* = 73, *z*‐value = 4.41, *T*‐value = 5.22, *p*_FWE_ < .05; see Figure 5b). This analysis revealed no other significant activations.

## 4 DISCUSSION

Despite being widely used, the behavioral and neural effects of nutrition claims on food perception and valuation are not well understood. To assess the effects of nutrition claims on expectations, perception, and valuation, we conducted two studies. In Study 1, we tested and compared the behavioral effects of a “fat‐reduced” claim with those of a “protein‐rich” claim. We found that both claims influenced only expected but not perceived taste; there were no differences between both claims in these effects. The “fat‐reduced” claim decreased expected and perceived satiation, whereas the “protein‐rich” claim increased only expected satiation. Both claims increased expectations and perceptions of healthiness, however, the “protein‐rich” claim increased healthiness expectations significantly more than the “fat‐reduced” claim, with no additional costs on pleasantness. In Study 2, we assessed whether the “protein‐rich” claim impacted perceived taste pleasantness, valuation, and their neural correlates. In this study, we replicated several of the findings from Study 1 and further found that the “protein‐rich” claim increased willingness to pay for otherwise equal drinks and was associated with an increased activity in left lateral OFC during cue viewing, a decreased activity in a cluster extending into left NAcc during tasting, and a decreased functional connectivity between the NAcc cluster and a cluster in right middle frontal gyrus during swallowing.

### 4.1 Behavioral results

#### 4.1.1 Effects of nutrition claims on expected and perceived taste pleasantness

In both studies, we found that nutrition claims influenced only expected but not perceived taste pleasantness. While previous studies have reported similar findings (Levin & Gaeth, 1988; Norton et al., 2013), others have reported that nutrition claims influence both expected and perceived pleasantness (Bialkova et al., 2016; Liem et al., 2012; Ng et al., 2011; Oostenbach et al., 2019; Piqueras‐Fiszman & Spence, 2015). These inconsistencies may be due to differences in claims, food products, sample characteristics, and combination of these factors across studies (Benson et al., 2018; Bialkova et al., 2016; Choi et al., 2012; Kaur et al., 2017).

While previous research has shown effects on perceived pleasantness in dieting, restrained eaters or individuals with obesity (Cavanagh & Forestell, 2013; Irmak et al., 2011; Ng et al., 2011; Wansink & Chandon, 2006), we tested hungry healthy participants with healthy eating styles. Either nutrition claims do not influence perceived pleasantness in healthy participants, or the effect is so small that we cannot assess it with our study design, with which we could exclude small‐to‐medium effect sizes according to Cohen's criteria (Cohen, 1988; Cohen's *d* ≥ 0.23 and *d* *≥* 0.34, for Studies 1 and 2, respectively). More powerful studies are needed to pursue this question.

As both claims decreased expected taste pleasantness but not perceived taste, they led to positive taste prediction errors. This finding, together with the fact that drink preference was explained by perceived rather than expected taste pleasantness, suggests that exposure may be a good strategy to update negative expectations associated with claims and perhaps increase their acceptance. Indeed, previous studies have shown that single and repeated exposure to certain stimuli, including food products, positively impact preference and acceptance of products (Appleton et al., 2018; Ballard et al., 2017; Zajonc, 1968). Future studies could investigate whether nutrition claims enhance or attenuate exposure effects.

We found that both the “protein‐rich” and the “fat‐reduced” claim had similar effects on expected and perceived pleasantness, suggesting that the type of the claim may be less relevant for pleasantness, and more relevant for other attributes. To our knowledge, there are no previous experimental studies comparing the effects of fat and protein claims like we did, therefore, these novel findings and our interpretation should be further explored in the future.

#### 4.1.2 Effects of nutrition claims on expected and perceived healthiness

In line with previous research we found that nutrition claims increased expectations and perceptions of healthiness (Benson et al., 2018; Chrysochou & Grunert, 2014; Oostenbach et al., 2019; Prada et al., 2021; Williams, 2005). Comparing the two nutrition claims, we found that the “protein‐rich” claim influenced expected healthiness more than the “fat‐reduced” claim. This observed difference aligns with the findings of André et al. (2019), who conducted several survey studies and found that healthiness increases for claims that are scientific and focus on the presence of a positive attribute (e.g., protein) rather than on the removal of a negative attribute (e.g., fat). Interestingly, we found that both claims increased perceived healthiness even after sampling otherwise equal drinks. Healthiness is considered as an abstract food attribute (Rangel, 2013) reflecting rather long‐term benefits of consuming a certain food, and it may require more cognitive rather than sensory processing. As such, it is conceivable that healthiness may not be influenced by a short‐term exposure to food, but is more strongly influenced by labels and claims.

#### 4.1.3 Effects of nutrition claims on expected and perceived satiating quality

Our findings are in line with previous research showing that reduced‐fat claims decrease expectations and perception of satiating quality (Chandon & Wansink, 2012; Faulkner et al., 2014; Wansink & Chandon, 2006). Different than the “fat‐reduced” claim, we found that the “protein‐rich” claim increased expected satiating quality; this is consistent with the fact that proteins are largely considered as satiating nutrients (Chambers et al., 2015). Interestingly, we found that perceived satiating quality for the same drinks changed depending on the claim with which they were presented: while the “protein‐rich” claim slightly increased the ratings, the “fat‐reduced” claim decreased them. These findings support the importance of labeling as a strategy targeting portion size control and satiation (Benson et al., 2018; Chambers et al., 2015; Gibson‐Moore, 2009; Van Kleef et al., 2012). In this context, our results suggest that including a “protein‐rich” claim increases expectations of satiating quality and may thus positively impact portion size selection.

Our exploratory analyses revealed gender differences in the effects of the “protein‐rich” and “fat‐reduced” claims on expected satiating quality. More specifically, in Study 1 we found that compared to men, women exhibited a higher claim effect on expected satiation for the “protein‐rich” claim compared to the “fat‐reduced” claim. Furthermore, in this study, the effect of the “protein‐rich” claim on expected satiation was higher for women than for men. We, however, could not replicate the effects of the “protein‐rich” claim in Study 2, suggesting these associations may not be as robust. Indeed, gender specific differences on nutrition claim effects have not been systematically reported in previous literature (Dean et al., 2007; Prada et al., 2021; Steinhauser & Hamm, 2018). Importantly, our studies were not designed to assess associations between gender and claim effects, and therefore our results should be considered accordingly. For instance, we did not counterbalance gender in Study 1, which resulted in a different number of men and women in each condition (Protein condition: 27 men, 30 women; Fat condition: 16 men, 37 women). The association between gender and nutrition claims effects on valuation should be clarified in future studies designed to specifically assess such effects.

#### 4.1.4 Effects of nutrition claims on willingness to pay

We found that participants were willing to pay more for protein‐rich drinks, which they expected to be less tasty, but healthier and more satiating, than for conventional drinks. This finding aligns with previous research showing that participants are willing to pay more for food they perceive as healthy, especially if it is presented with labels that highlight their nutritious value (Enax et al., 2015a). Previous research has shown that salient nutrition labels impact valuation by decreasing the weight of taste and increasing the weight of healthiness in food decisions (Enax et al., 2016; Rramani et al., 2020). Along these lines, we found that while for conventional drinks WTP ratings were explained only by expected taste pleasantness ratings, for protein‐rich drinks they were explained only by expected satiation ratings. These results may indicate that the presence of nutrition claims may decrease the impact of taste and increase the impact of other attributes such as expected satiety on valuation. Such effects could be more specifically tested in future studies. Importantly, we assessed WTP only before sampling the drinks, not during or after. Therefore, we cannot conclude whether exposure to the taste and flavor of drinks impacts participants’ WTP. Future studies including a WTP measure after and/or during every sampling should assess these effects more specifically.

### 4.2 fMRI results

Our fMRI task elicited responses in regions previously implicated in taste and flavor processing such as bilateral caudate, orbital part of the inferior frontal gyrus, insula, frontal operculum, and anterior cingulate cortex (Avery et al., 2020; de Araujo et al., 2003; Grabenhorst et al., 2008; van Rijn et al., 2018; Veldhuizen et al., 2011). These results support the notion that our fMRI task could reliably evoke and measure taste and flavor responses in the brain.

We found an increased activity in left lateral OFC when viewing cues for drinks that were expected to be less tasty, but healthier, and more satiating (“protein‐rich” drinks). Lateral OFC is associated with evaluation of taste pleasantness (Bender et al., 2009; Kringelbach et al., 2003) and inhibition of rewarding responses (Elliott, 2000; Kringelbach, 2005; van der Laan et al., 2014) and supports representations of the nutritive attributes of food (Suzuki et al., 2017) and their healthiness (Londerée & Wagner, 2021). Thus, claim effects on lateral OFC activation may reflect changes in the representation of food items, which were revealed in participants’ expectations. This hypothesis is consistent with previous findings from Courtney et al. (2018) who showed that providing caloric information on food images alters the representation of these food items in lateral OFC. Future studies using multivariate approaches may be more suitable to investigate the changes in neural representation of food items by claims.

We found no difference in neural activity in regions associated with valuation (vS/NAcc, vmPFC, dlPFC, lateral OFC) when tasting nor when swallowing drinks presented with *vs*. without the “protein‐rich” claim. By contrast, we found an increased activity in a cluster extending into the left NAcc when tasting drinks presented without *vs*. with the “protein‐rich” claim, despite no difference in taste pleasantness ratings at the behavioral level. NAcc is among other functions involved in reward anticipation (Berridge et al., 2010; Knutson et al., 2008; O'Doherty et al., 2002; Small et al., 2008). As drinks without claim were expected to be less healthy but tastier, our finding might reflect the expectation of a better taste of drinks without the claim. Such an interpretation is also concordant with literature on placebo effects supporting that vS/NAcc activity may reflect motivational aspects associated with a certain stimulus, rather than its rewarding properties. In other words, an increased vS/NAcc activity may reflect participants’ wanting to believe or expect that a certain stimulus is better than others (Wager & Atlas, 2015; Schmidt et al., 2017). Furthermore, considering that drinks without claims were expected to be not only tastier but also less healthy, the increased activity in left NAcc when tasting drinks without *vs*. with claims partially supports the unhealthy‐tasty intuition (Raghunathan et al., 2006), whereby healthier food is expected to be less tasty. Contrary to this intuition, however, higher healthiness expectations did not negatively impact reported perceived taste pleasantness.

Previous research has shown that context‐dependent beliefs and expectations often override experienced pleasantness (Plassmann et al., 2008; Schmidt et al., 2017; Wager & Atlas, 2015). We could not replicate such effects with nutrition claims, possibly because claim‐induced expectations may not have been as strong and/or they may have been “updated” once participants were exposed to even more sensory characteristics of the stimuli. Such an explanation is likely considering that we did not find an increased activity in left NAcc during swallowing drinks but only during tasting them. During swallowing, we found an increased task‐dependent functional connectivity between the cluster extending into the left NAcc and a cluster of the right middle frontal gyrus. Middle frontal gyrus is a region involved in successful action cancelation (Dambacher et al., 2014), in processing conflicting information and error monitoring (Suárez‐Pellicioni et al., 2013), and in reorienting attention from endogenous (goal‐driven, top‐down) to exogenous (stimulus‐driven, bottom‐up) control (Chica et al., 2013; Corbetta et al., 2008; Japee et al., 2015). Considering this, it is possible that when additional sensory information becomes available (e.g., flavor), attention may be redirected to the perceived stimulus characteristics thereby “updating” expectations or reducing their effects on perceived pleasantness. To our knowledge, right middle frontal gyrus has not been commonly associated with contextual effects on taste valuation in past research; therefore, this finding and its interpretation should be validated in future studies.

Altogether our fMRI findings suggest that when less sensory information is present, during cue viewing and tasting as opposed to flavoring, expectations may modulate neural activity associated with reward processing. However, once additional sensory information becomes available, bottom‐up processes may contribute to updating expectations or reducing their effect on valuation. This interpretation implies that while nutrition claims may initially induce a top‐down bias on valuation through the expectations that they elicit, this is not sustained when additional sensory information becomes available, possibly due to attention reorientation processes that may relate to stimuli re‐evaluation. Future studies should examine these effects more closely.

### 4.3 Limitations and suggestions for future research

Our studies have limitations which could be considered in future research. First, the way we assessed perceived satiating quality may not be ideal, especially since many satiety signals may not arise immediately at the moment of consumption (Ahima & Antwi, 2008; Chambers et al., 2015; Wright et al., 2016). Measuring hormones in the blood (e.g., ghrelin like in Crum et al., 2011) may be a better measure of satiety that could be considered in future studies. Second, even though in both our studies we used protein‐rich and fat‐reduced milk‐mix drinks, we did not present the drinks with the two nutrition claims at once. Considering that many foods, especially novel functional foods, contain multiple claims on their packaging, it is relevant to assess the effects of different nutrition claims presented together. Third, we assessed WTP only before sampling the drinks, so we could not assess claim effects on WTP after exposure. Furthermore, our WTP measure was hypothetical. Different than incentivized WTP measures, hypothetical WTP measures do not have a tangible and real consequence for the participants and may therefore lead to overestimated values, even though they are generally reported to be valid and efficient in assessing subjective preference (Schmidt & Bijmolt, 2020). When using incentivized measures, it is more likely that participants incorporate longer‐term consequences in valuation, since their behavior impacts the outcome. By contrast, in our study, independent of their ratings, participants had to taste the different drinks. Not using incentivized measures may explain why we did not find an effect of nutrition claims on the activity of regions associated with integration of longer‐term consequences in valuation such as dlPFC (Enax et al., 2015a; Schmidt et al., 2017). Future studies on the effects of nutrition claims should consider using incentivized measures and assess valuation pre‐ and post‐exposure to certain stimulus characteristics. Finally, while our fMRI study was adequately powered to reveal taste and flavor responses in the brain, it may not have been sufficiently powerful to detect smaller effects. While the effects of claims on expectations are robust, the effects of these expectations on perceived pleasantness are likely smaller and may be detected only in more powerful studies requiring larger sample sizes.

## 5 CONCLUSION

In our two studies we found that nutrition claims impacted expectations of taste pleasantness, healthiness, and satiating quality, but did not impact perceived taste pleasantness. We found that the “protein‐rich” claim increased healthiness expectations significantly more than the “fat‐reduced” claim, at no additional cost on expected and perceived pleasantness. Our fMRI results suggest that while claim‐induced expectations may modulate reward‐associated responses during cue viewing and tasting otherwise equal drinks, such effects are not sustained during swallowing these drinks. Our results support two strategies that could increase acceptance for foods with nutrition claims. First, with our studies we provide experimental evidence supporting that higher healthiness expectations do not negatively impact perceived taste pleasantness. Considering this, it may be more efficient to use nutrition claims that elicit higher healthiness expectations, especially claims that highlight the presence of nutrients with more positive associations. Second, we found that even though nutrition claims elicited negative taste pleasantness expectations, exposure to foods with such claims positively surprised participants and impacted their preference. This indicates that exposing participants to foods with nutrition claims may modulate their negative expectations and even increase acceptance for such foods. Overall, our studies provide novel insights into the effects of two different nutrition claims, especially the “protein‐rich” claim, on expected and perceived attributes of the same food and point out possible novel neural correlates of nutrition claim effects on expectations during tasting and swallowing otherwise equal drinks.

## CONFLICT OF INTEREST

The authors declare no conflicts of interest.

### ETHICS STATEMENT

The study was approved by the local ethical committee of the University of Bonn, Medical Center (No. 077/19) and all participants provided written informed consent according to the declaration of Helsinki.

### PEER REVIEW

The peer review history for this article is available at: https://publons.com/publon/10.1002/brb3.2828.

## Supporting information

Supplementary Table S1. Descriptive statistics and between‐group comparison of age, gender, and baseline characteristics in Study 1
Supplementary Table S2. Association between gender and claim effects on expected and perceived attributes in Study 1
Supplementary Table S3. Probability of preferring a drink presented with a nutrition claim in Study 1
Supplementary Figure S1. Claim effects on expected and perceived attributes for men and women in Study 1.
Supplementary Figure S2. Taste pleasantness, healthiness, and satiating quality prediction errors for fat‐claim and protein‐claim condition in Study 1.
Supplementary Table S4. Descriptive statistics for the administered questionnaires and the sweet taste sensitivity in Study 2
Supplementary Table S5 Relation between expectations and willingness to pay for protein‐rich and conventional drinks in Study 2
Supplementary Table S6. Association between gender and claim effects on expected attributes, perceived taste pleasantness, and willingness to pay in Study 2
Supplementary Table S7. Effect of expectations and perceived taste pleasantness on the probability of preferring a drink with the “protein‐rich” claim in Study 2
Supplementary Table S8. Small volume correction analyses results
Supplementary Figure S3. Common masks including regions of interest associated with taste (A.) and valuation (B.) processing used for small volume correction analyses. (A.)
Supplementary Figure S4. Taste pleasantness prediction errors in Study 2.
Supplementary Figure S5. Brain activations for tasting (A.) and swallowing (B.) drinks *vs*. rinsing solution.
Supplementary Figure S6. The midbrain cluster more active when tasting drinks without *vs*. with the “protein‐rich” claim (shown in red) extends into the left nucleus accumbens (shown in blue) and to a small degree into the left pallidum (shown in green).
Supplementary Table X. fMRI data quality measures.

## Data Availability

The behavioral data, the group level fMRI contrast parameter maps, and ROI masks are openly available in the Harvard Dataverse at: https://dataverse.harvard.edu/privateurl.xhtml?token=3373c390‐d771‐4bd8‐9470‐d8ab356874c0.

## Footnotes

1 “Health halo” effects occur when the perceived and/or expected healthiness of a product generalizes to the other qualities of that product and discourages consumers to seek further information about these other qualities or characteristics. For instance, a low‐fat product may be considered as healthier but also as having less calories by default, even though this may not always be the case.

2 This information was given in written form as part of the instructions, and was taken from the “Regulation on nutrition and health claims made on foods” adopted by the EU in December 2006 (European Union Parliament, 2006; regulation [EC] No 1924/2006). According to this regulation, foods containing the “protein‐rich” claim (or any other protein claim likely to have the same meaning for the consumer) must contain at least 30% more protein than a comparable regular reference product, and at least 20% of the energy value of these foods must come from proteins; foods containing the “fat‐reduced”/“low‐fat” claim must contain at least 30% less fat than a comparable regular reference product (<3g/100g).
