# Supplementary material for: Nutrition claims influence expectations about food attributes, attenuate activity in reward‐associated brain regions during tasting, but do not impact pleasantness
Source: Brain Behav. 2022 Dec 13;13(1):e2828. doi: 10.1002/brb3.2828 (PMC9847625; doi:10.1002/brb3.2828)
Supplement: Supplementary file 1 — Supplementary Table S1. Descriptive statistics and between‐group comparison of age, gender, and baseline characteristics in Study 1 Supplementary Table S2. Association between gender and claim effects on expected and perceived attributes in Study 1 Supplementary Table S3. Probability of preferring a drink presented with a nutrition claim in Study 1 Supplementary Figure S1. Claim effects on expected and perceived attributes for men and women in Study 1. Supplementary Figure S2. Taste pleasantness, healthiness, and satiating quality prediction errors for fat‐claim and protein‐claim condition in Study 1. Supplementary Table S4. Descriptive statistics for the administered questionnaires and the sweet taste sensitivity in Study 2 Supplementary Table S5 Relation between expectations and willingness to pay for protein‐rich and conventional drinks in Study 2 Supplementary Table S6. Association between gender and claim effects on expected attributes, perceived taste pleasantness, and willingness to pay in Study 2 Supplementary Table S7. Effect of expectations and perceived taste pleasantness on the probability of preferring a drink with the “protein‐rich” claim in Study 2 Supplementary Table S8. Small volume correction analyses results Supplementary Figure S3. Common masks including regions of interest associated with taste (A.) and valuation (B.) processing used for small volume correction analyses. (A.) Supplementary Figure S4. Taste pleasantness prediction errors in Study 2. Supplementary Figure S5. Brain activations for tasting (A.) and swallowing (B.) drinks vs. rinsing solution. Supplementary Figure S6. The midbrain cluster more active when tasting drinks without vs. with the “protein‐rich” claim (shown in red) extends into the left nucleus accumbens (shown in blue) and to a small degree into the left pallidum (shown in green). Supplementary Table X. fMRI data quality measures. [file BRB3-13-e2828-s001.docx]

Supplementary Material

1. STUDY 1 (BEHAVIORAL STUDY)

Supplementary Table S1. Descriptive statistics and between-group comparison of age, gender, and baseline characteristics in Study 1

|  | **Protein condition**  **(*n* = 57)** | **Fat condition**  **(*n* = 53)** | **Statistical Comparison** |
| --- | --- | --- | --- |
| Gender | 30 Women, 27 Men | 37 Women, 16 Men | *χ^2^*_(1)_ = 2.72, *p* = 0.10 |
|  | ***M (SD)*** | ***M (SD)*** |  |
| Age | 23.79 *(3.51)* | 23.53 *(2.97)* | *t*_(107.1)_ = –0,42, *p* = 0.674 |
| Body Mass Index | 23.83 *(2.48)* | 24.35 *(2.29)* | *t*_(108)_ = 1.15, *p* = 0.254 |
| **Baseline status** |  |  |  |
| Hunger | 5.53 *(2.16)* | 5.30 *(2.47)* | *t*_(103.68)_ = –0.50, *p* = 0.614 |
| Arousal/Excitement | 3.56 *(1.70)* | 3.81 *(2.04)* | *t*_(101.59)_ = 0.69, *p* = 0.488 |
| Valence/Satisfaction | 6.63 *(1.29)* | 6.30 *(1.81)* | *t*_(93.26)_ = –1.09, *p* = 0.278 |
| Hours of Sleep | 7.58 *(0.80)* | 7.54 *(0.77)* | *t*_(107.84)_ = 0.27, *p* = 0.784 |

*Note:* Independent sample *t*-tests were used to assess group differences for continuous variables, whereas a Chi-square test was used to compare the frequencies of men and women in both groups. *M:* mean, *SD*: standard variation.

Supplementary Table S2. Association between gender and claim effects on expected and perceived attributes in Study 1

|  | **DV: Expected taste pleasantness** | | | **DV: Expected healthiness** | | | **DV: Expected satiating quality** | | |
| --- | --- | --- | --- | --- | --- | --- | --- | --- | --- |
| **Fixed effects** | **B (*SE*)** | **95% CI** | ***p*** | **B (*SE*)** | **95% CI** | ***p*** | **B (*SE*)** | **95% CI** | ***p*** |
| Intercept | –0.97 (*0.32*) | [–1.61, –0.33] | 0.003 | 0.05 (*0.22*) | [–0.38, 0.49] | 0.81 | –0.59 (*0.21*) | [–1.02, –0.17] | 0.01 |
| Gender  (1 = Man, 0 = Woman) | 0.29 (*0.59*) | [–0.88, 1.45] | 0.63 | 0.82 (*0.40*) | [0.02, 1.62] | 0.04 | 0.09 (*0.39*) | [–0.68, 0.87] | 0.81 |
| Condition  (1 = Protein, 0 = Fat) | –0.29 (*0.48*) | [–1.25, 0.66] | 0.54 | 0.68 (*0.33*) | [0.02, 1.34] | 0.04 | 2.09 (*0.32*) | [1.46, 2.73] | < 0.001 |
| Gender x Condition | –0.17 (*0.78*) | [–1.72, 1.39] | 0.83 | –0.44 (*0.54*) | [–1.51, 0.62] | 0.41 | –1.08 (*0.52*) | [–2.11, –0.04] | 0.04 |
| **Model** |  |  |  |  |  |  |  |  |  |
| R2/R2 adjusted | 0.010/–0.018 | | | 0.093/0.068 | | | 0.004/–0.024 | | |
|  | **DV: Perceived taste pleasantness** | | | **DV: Perceived healthiness** | | | **DV: Perceived satiating quality** | | |
| **Fixed effects** | **B (*SE*)** | **95% CI** | ***p*** | **B (SE)** | **95% CI** | ***p*** | **B (SE)** | **95% CI** | ***P*** |
| Intercept | 0.20  (*0.23*) | [–0.26, 0.67] | 0.39 | 0.26 (*0.17*) | [–0.08, 0.60] | 0.14 | –0.49 (*0.22*) | [–0.92, –0.05] | 0.03 |
| Gender  (1 = Man, 0 = Woman) | –0.14 (*0.43*) | [–0.99, 0.70] | 0.74 | 0.52 (*0.31*) | [–0.10, 1.15] | 0.10 | 0.14 (*0.40*) | [–0.65, 0.93] | 0.72 |
| Condition  (1 = Protein, 0 = Fat) | –0.22 (*0.35*) | [–0.91, 0.47] | 0.53 | 0.39 (*0.26*) | [–0.12, 0.90] | 0.13 | 0.75 (*0.33*) | [0.11, 1.40] | 0.02 |
| Gender x Condition | 0.21 (*0.57*) | [–0.92, 1.34] | 0.71 | –0.47 (*0.42*) | [–1.30, 0.36] | 0.26 | –0.21 (*0.53*) | [–1.26, 0.85] | 0.70 |
| **Model** |  |  |  |  |  |  |  |  |  |
| R2/R2 adjusted | 0.004/–0.024 | | | 0.041/0.014 | | | 0.065/0.038 | | |

Notes: Effects are estimated using linear regression models. DV: dependent variable; B: unstandardized estimate; SE = standard error of the estimate; CI: confidence interval; n_Men_ = 43 (Fat =16; Protein = 27); n_Women_ = 67 (Fat = 37, Protein = 30).

Supplementary Table S3. Probability of preferring a drink presented with a nutrition claim in Study 1

|  | **DV: Preferred drink with a nutrition claim** | | | | | | | | |  |
| --- | --- | --- | --- | --- | --- | --- | --- | --- | --- | --- |
| **Fixed effects** | ***OR (SE)*** | **95% CI** | ***p*** | ***OR (SE)*** | **95% CI** | ***p*** | ***OR (SE)*** | **95% CI** | ***p*** | |
| Intercept | 1.34 (*0.42*) | [0.73, 2.49] | 0.339 | 1.22 (*0.43*) | [0.62, 2.45] | 0.572 | 1.02 (*0.30*) | [0.56, 1.84] | 0.958 | |
| Condition Protein  (1 =Yes, 0 = No) | 1.09 (*0.50*) | [0.44, 2.70] | 0.852 | 1.07 (*0.52*) | [0.41, 2.81] | 0.895 | 0.93 (*0.38*) | [0.41, 2.10] | 0.866 | |
| Expected Taste Pleasantness Difference | 1.09 (*0.11*) | [0.89, 1.34] | 0.410 |  |  |  |  |  |  | |
| Expected Healthiness Difference | 0.99 (*0.14*) | [0.74, 1.32] | 0.937 |  |  |  |  |  |  | |
| Expected Satiating Quality Difference | 0.92 (*0.13*) | [0.68, 1.22] | 0.548 |  |  |  |  |  |  | |
| Perceived Taste Pleasantness Difference |  |  |  | 3.30 (*0.87*) | [2.07, 5.87] | < 0.001 |  |  |  | |
| Perceived Healthiness Difference |  |  |  | 0.95 (*0.22*) | [0.60, 1.50] | 0.838 |  |  |  | |
| Perceived Satiating Quality Difference |  |  |  | 1.01 (*0.20*) | [0.69, 1.48] | 0.978 |  |  |  | |
| Taste Pleasantness Prediction Error |  |  |  |  |  |  | 1.30 (*0.14*) | [1.07, 1.62] | 0.012 | |
| Healthiness Prediction Error |  |  |  |  |  |  | 0.99 (*0.15*) | [0.73, 1.35] | 0.927 | |
| Satiating Quality Prediction Error |  |  |  |  |  |  | 1.09 (*0.12*) | [0.88, 1.36] | 0.427 | |
| **Model** |  |  |  |  |  |  |  |  |  | |
| R^2^ Tjur | 0.010 |  |  | 0.289 |  |  | 0.076 |  |  | |

Note: Effects are estimated using logistic regression. R^2^ Tjur is a type of pseudo R^2^ that indicates the distance between two means for the predicted values. The closer this value gets to 1, the clearer the separation between the predicted values for 0 and 1. All differences are calculated by subtracting the ratings for the drink presented without a claim, from the ratings of the drinks without a claim. Note that differences in prediction errors are entered in a separate model, because as mentioned previously they are simply the difference between perceived and expected qualities and thus correlate highly with these ratings. DV: dependent variable; OR: odds ratio; SE = standard error; CI: confidence interval. n_Protein_ = 57, n_Fat_ = 53.


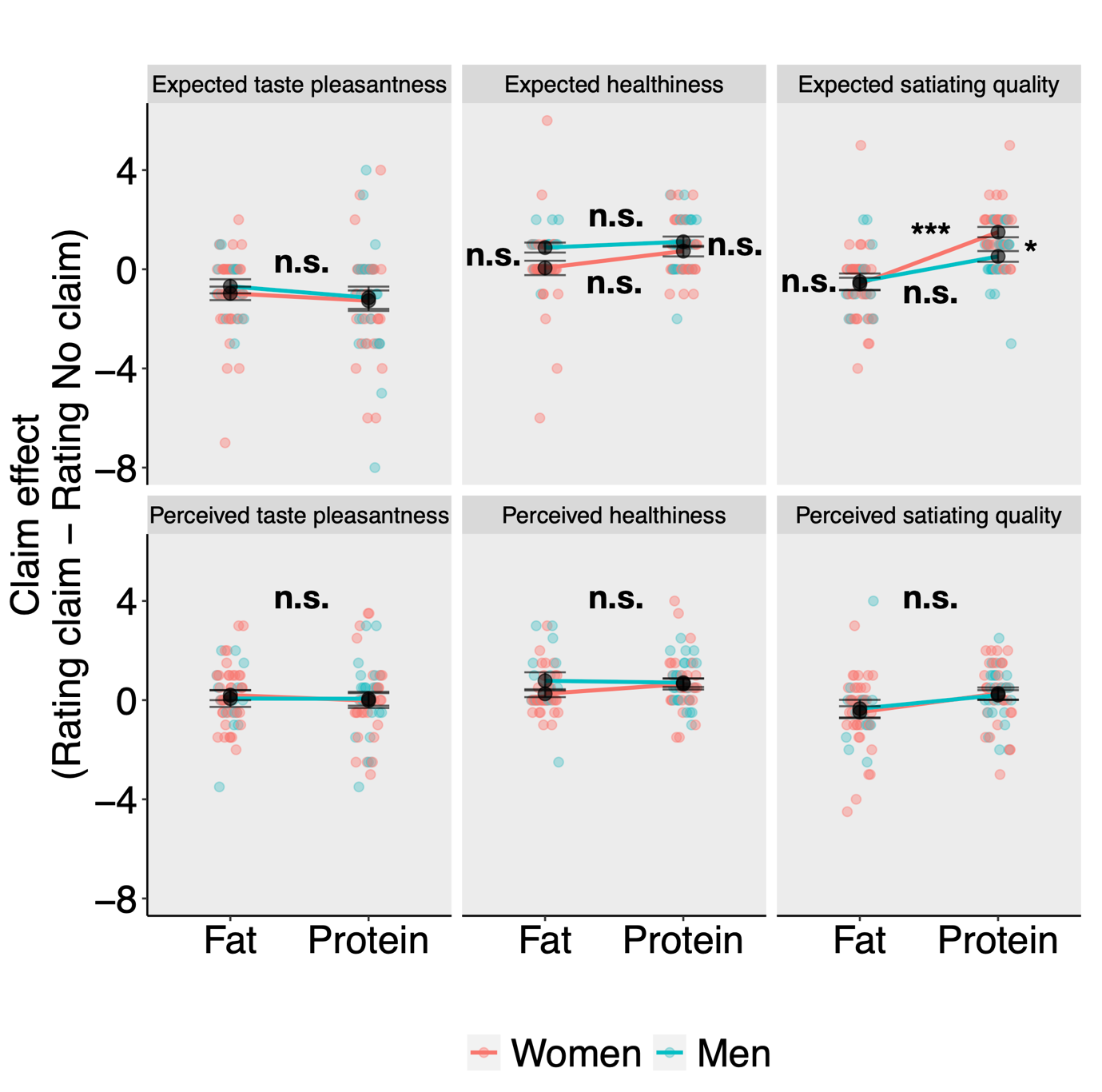
Supplementary Figure S1. Claim effects on expected and perceived attributes for men and women in Study 1. Colorful dots are individual mean values for women (red) and men (turquoise), whereas dark gray dots are mean values across conditions. Post-hoc Tukey tests were used for pairwise comparisons. Error bars represent the standard error of the mean; *n*_Men_ = 43 (Fat =16; Protein = 27); *n*_Women_ = 67 (Fat = 37, Protein = 30); ****p* < 0.001, **p* < 0.05, n.s.: not significant.

**Supplementary Figure S2. Taste pleasantness, healthiness, and satiating quality prediction errors for fat-claim and protein-claim condition in Study 1.** Differences are calculated by subtracting the expected claim effect from the perceived claim effect. One-sample t-tests were used to assess whether each prediction error was different from zero, whereas two-sample t-tests (values shown in graph) were used to compare prediction errors between groups. Gray dots are individual prediction errors. Black dots are mean values across participants, and error bars represent the standard error of the mean; n_Protein_ = 57, n_Fat_ = 53; *** p < 0.001, ** p < 0.01, n.s.: not significant.

1. STUDY 2 (FMRI STUDY)
   1. Supplementary Methods
      1. Rinsing solution preparation

In Study 2 (fMRI study), we used a rinsing solution delivered at the end of each trial, to avoid spillover over trials, and to provide a baseline condition which can later be used to assess whether there is at all a taste response that can be captured at the neural level. Considering that water is not tasteless (Bartoshuk, 1968; de Araujo et al., 2003) and is therefore not a good control solution, as a rinsing solution we used different concentrations of a solution that resembles human saliva. We followed the same procedure as in Seubert et al. (2015). We prepared a water-based stock solution of 2.5mM sodium bicarbonate and 24mM potassium chloride, and then made 25, 50, 75, and 100% concentrations of this solution. Before the fMRI experiment, we presented these four concentrations to every participant and asked them to indicate which of these they found to be the most “tasteless”; this concentration was then used as a rinsing solution in the fMRI task. We prepared fresh rinsing solutions every second day and stored them refrigerated at 4°C.

- - 1. Sweet taste sensitivity test procedure

In Study 2, we used a sweet taste sensitivity test to assess participants’ ability to taste sweetness. For this, we estimated sucrose recognition thresholds using an adaptive procedure based on QUEST+ (Watson, 2017), which is an extension of an established protocol using a yes-no task (Höchenberger and Ohla, 2017, 2019). The procedure was implemented to run in a web-browser. During the test, participants were blindfolded to minimize distraction and asked to extend their tongue. A taste stimulus (approx. 0.5 ml sucrose solution) was then dropped with a pipette onto the anterior tongue by the experimenter. Participants’ task was to indicate whether the stimulus tastes sweet or not while the tongue was still protruded. The trial was repeated if the subject retracted the tongue before providing an answer. The inter-stimulus interval was approximately 30 sec and participants rinsed their mouths with distilled water before a new trial. The measurement concluded if the 5% to 95 % confidence interval was smaller than half the concentration presented last (dynamic termination criterion; see Höchenberger and Ohla, 2019) with the constraints that each measurement be based on at least 10 and at most 20 trials.

For the taste sensitivity test, we used sucrose solutions. For this, sucrose (Sigma-Aldrich, CAS number: 57-50-1) was dissolved in distilled water to achieve 17 equidistantly spaced concentrations on a decadic logarithmic grid ranging from 0.001 mM to 0.5623 mM. Taste solutions were stored refrigerated at 4°C for a maximum duration of seven days in glass bottles and presented at room temperature during testing.

- - 1. Psycho Physiological Interaction (PPI) analysis

To test if the “protein-rich” claim impacted functional connectivity patterns in the brain, we conducted PPI analyses using the *gPPI* toolbox (McLaren et al., 2012). For this analysis, we first estimated a 1^st^ level GLM in which we included 23 regressors: two for cue (claim, no claim), two for tasting (claim, no claim), two for swallowing (claim, no claim), rating, rinsing, swallow rinsing, two for PPI*cue (claim, no claim), two for PPI*tasting (claim, no claim), two for PPI*swallowing (claim, no claim), seed time series, six movement regressors (three rotation, three translation), and a constant. We estimated this model for every participant and for each participant we calculated six contrasts: PPI* cue claim *vs.* PPI*cue no claim and *vice versa*, PPI*tasting claim *vs.* PPI*tasting no claim and *vice versa*, and PPI*swallowing claim *vs.* PPI*swallowing no claim and *vice versa*. These calculated contrasts were subjected to one-sample *t*-tests (2^nd^ level analyses). We performed this analysis for every region that was associated with a claim effect in the univariate analyses and looked at functional connectivity changes between these seed regions and the whole brain.

- 1. Supplementary Results
     1. fMRI Data Quality Check

Prior to analyzing the neuroimaging data acquired in Study 2, we checked the quality of the obtained functional and structural images. We visually inspected all the functional and structural images, extracted and inspected movement parameters for every participant, and additionally used MRIqc (Esteban et al., 2017) to obtain more objective quality measures. In addition to looking at different quality measures, we also estimated effects of interest contrasts (*F*-contrast). Visual inspections of these contrasts indicated that for all participants there was a task-related activity that showed variation across the brain; we did not exclude any participant based on these contrasts. After exclusion was applied all the remaining datasets had no further identifiable issues. For a summary of the different quality measures obtained from MRIqc, see **Supplementary Table X** below.

Supplementary Table X. fMRI data quality measures

|  |  |  |  | **Max. translations (mm)** | | | **Max. rotations (mm)** | | | |  |
| --- | --- | --- | --- | --- | --- | --- | --- | --- | --- | --- | --- |
| **ID** | **Volumes** | **SNR median** | **tSNR median** | **x** | **y** | **z** | | **x** | **y** | **z** | |
| 1 | 717.00 | 5.15 | 38.01 | 0.2 | 0.5 | 0.7 | | 0.2 | 0.02 | 0.01 | |
| 2 | 692.00 | 5.08 | 34.43 | 0.001 | 0.2 | 0.9 | | 0.01 | 0.015 | 0.015 | |
| 3 | 701.00 | 5.49 | 31.04 | 0.3 | 0.2 | 2 | | 0.03 | 0.009 | 0.001 | |
| 4 | 718.00 | 5.73 | 36.82 | 0.3 | 0.3 | 1.1 | | 0.023 | 0.02 | 0.01 | |
| 5 | 703.00 | 5.45 | 38.42 | 0.2 | 0.8 | 1.1 | | 0.015 | 0.001 | 0.015 | |
| 6 | 723.00 | 4.92 | 30.51 | 0.3 | 1.2 | 2.2 | | 0.03 | 0.03 | 0.03 | |
| 7 | 692.00 | 6.22 | 34.56 | 0.2 | 1.2 | 1 | | 0.04 | 0.009 | 0.01 | |
| 8 | 700.00 | 5.36 | 26.69 | 0.5 | 2 | 2.7 | | 0.05 | 0.022 | 0.012 | |
| 9 | 703.00 | 5 | 31.08 | 0.5 | 0.8 | 0.8 | | 0.04 | 0.01 | 0.009 | |
| 10 | 710.00 | 4.53 | 32.24 | 0.18 | 0.23 | 0.8 | | 0.04 | 0.001 | 0.01 | |
| 11 | 732.00 | 5.63 | 29.54 | 0.1 | 0.3 | 2.7 | | 0.016 | 0.005 | 0.001 | |
| 12 | 704.00 | 5.84 | 33.52 | 0.5 | 0.2 | 1.5 | | 0.016 | 0 010 | 0.001 | |
| 13 | 711.00 | 6.22 | 30.62 | 0.3 | 1 | 2 | | 0.026 | 0.005 | 0.005 | |
| 14 | 708.00 | 5.2 | 25.39 | 0.001 | 0.5 | 1.6 | | 0.032 | 0.003 | 0.002 | |
| 15 | 746.00 | 6.08 | 35.06 | 0.5 | 0.52 | 1.7 | | 0.032 | 0.013 | 0.003 | |
| 16 | 701.00 | 6.93 | 35.76 | 0.4 | 0.5 | 2.5 | | 0.025 | 0.009 | 0.01 | |
| 17 | 737.00 | 5.78 | 39.97 | 0.4 | 0.8 | 0.5 | | 0.006 | 0.015 | 0.013 | |
| 18 | 724.00 | 6.25 | 36.05 | 0.8 | 0.3 | 1.3 | | 0.02 | 0.023 | 0.05 | |
| 19 | 692.00 | 5.89 | 32.09 | 0.7 | 0.5 | 1.5 | | 0.022 | 0.02 | 0.032 | |
| 20 | 699.00 | 5.53 | 34.9 | 0.02 | 0.01 | 2.1 | | 0.032 | 0.001 | 0.001 | |
| 21 | 682.00 | 5.89 | 35.09 | 0.04 | 0.1 | 2.5 | | 0.027 | 0.012 | 0.03 | |
| 22 | 678.00 | 5.81 | 38.73 | 0.5 | 0.6 | 1 | | 0.013 | 0.015 | 0.005 | |
| 23 | 698.00 | 6.41 | 41.46 | 0.3 | 0.4 | 0.8 | | 0.01 | 0.022 | 0.015 | |
| 24 | 742.00 | 6.73 | 29.23 | 0.5 | 0.7 | 2.2 | | 0.033 | 0.002 | 0.005 | |
| 25 | 705.00 | 6.24 | 32.87 | 0.3 | 0.1 | 1.5 | | 0.01 | 0.005 | 0.021 | |
| 26 | 705.00 | 5.25 | 31.23 | 0.5 | 0.7 | 1.5 | | 0.03 | 0.01 | 0.015 | |
| 27 | 681.00 | 5.8 | 28.82 | 0.3 | 0.5 | 2 | | 0.05 | 0.001 | 0.01 | |
| 28 | 723.00 | 7.11 | 36.42 | 0.2 | 0.7 | 1.9 | | 0.022 | 0.005 | 0.001 | |
| 29 | 712.00 | 6.33 | 38.85 | 0.3 | 1.3. | 1 | | 0.032 | 0.01 | 0.012 | |
| 30 | 728.00 | 6.1 | 34.48 | 0.5 | 0.5 | 0.27 | | 0.032 | 0.012 | 0.009 | |
| 31 | 754.00 | 6.23 | 37.36 | 0.2 | 0.5 | 0.2 | | 0.04 | 0.03 | 0.001 | |
| 32 | 707.00 | 5.59 | 30.29 | 0.2 | 0.9 | 1.2 | | 0.04 | 0.012 | 0.001 | |
| 33 | 763.00 | 5.21 | 35.08 | 0.1 | 0.2 | 1.6 | | 0.012 | 0.007 | 0.013 | |

ID: participant ID; Volumes: number of acquired functional volumes per participant; SNR: signal to noise ratio; tSNR: temporal signal to noise ratio. SNR and tSNR values are shown as calculated with MRIqc. Maximum translation and rotation is the maximum difference between an image and the first functional image of every participant.

- - 1. Sweet taste sensitivity test results

In our sample, sweet taste thresholds were numerically lower, than in previous reports that used a similar procedure based on QUEST (Hardikar et al., 2017; Höchenberger and Ohla, 2017, 2019). While it is difficult to unequivocally determine the cause of the apparent differences between studies, it seems plausible that two novel methodological aspects contributed to them: the consideration of response bias via “catch” trials in the QUEST+ algorithm (which is not part of the previously used QUEST) and the use of the dropping technique in contrast to the previously used spraying technique.

To assess the reliability of the measured sweet taste threshold, we looked at whether they relate to preference as reported in previous studies (Chamoun et al., 2019; Höchenberger and Ohla, 2019). To this end, we computed correlations between the sweet taste threshold and preference for sweet, salty, sour, and bitter food. We found no significant correlations between threshold and any of the preferences (all *p* > 0.20), even though the direction of their relations was similar to previous findings: preference for sweet taste relates negatively (r = –0.11), whereas preference for salty (*r* = 0.19), bitter (*r* = 0.20), and sour food (*r* = 0.11) relates positively with the assessed sensitivity thresholds (Hardikar et al., 2017; Chamoun et al., 2019).

- - 1. Supplementary Tables

Supplementary Table S4. Descriptive statistics for the administered questionnaires and the sweet taste sensitivity in Study 2

| **Measure** | **Min.** | **Max.** | **Median** | ***M*** | ***SD*** |
| --- | --- | --- | --- | --- | --- |
| **DEBQ questionnaire** |  |  |  |  |  |
| Restrained eating | 1.1 | 4.10 | 2.00 | 2.23 | 0.84 |
| External eating | 1.7 | 4.60 | 3.30 | 3.36 | 0.67 |
| Emotional eating | 1.0 | 5.0 | 2.00 | 2.30 | 0.93 |
| **Baseline status** |  |  |  |  |  |
| Hunger | 3 | 8 | 6 | 5.97 | 1.33 |
| Arousal/Excitement | 1 | 8 | 4 | 4.08 | 1.48 |
| Valence/Satisfaction | 2 | 9 | 7 | 6.72 | 1.36 |
| Hours of Sleep | 3 | 10 | 7.5 | 7.27 | 1.31 |
| Perceived Stress | 1 | 7 | 3 | 3.03 | 1.58 |
| **Sweet taste test sensitivity** | | | |  |  |
| Threshold (log 10 mol/L) | –2.90 | –0.37 | –1.30 | –1.29 | 0.51 |
| Slope | 2.60 | 3.25 | 3.03 | 2.98 | 0.17 |
| Lower Asymptote | 0.05 | 0.15 | 0.09 | 0.10 | 0.04 |

M: mean; SD: standard deviation; *n* = 39.

Supplementary Table S5 Relation between expectations and willingness to pay for protein-rich and conventional drinks in Study 2

|  | **DV: WTP for protein-rich drinks** | | | **DV: WTP for conventional drinks** | | |
| --- | --- | --- | --- | --- | --- | --- |
| **Fixed effects** | **B *(SE)*** | **95% CI** | ***p*** | **B *(SE)*** | **95% CI** | ***p*** |
| Intercept | 1.53 *(0.10)* | [1.33, 1.72] | < 0.001 | 1.37 *(0.10)* | [1.17, 1.57] | < 0.001 |
| Expected Taste Pleasantness | –0.13 *(0.11)* | [–0.35, 0.10] | 0.265 | 0.21 *(0.10)* | [0.004, 0.42] | 0.046 |
| Expected Healthiness | –0.03*(0.11)* | [–0.25, 0.19] | 0.782 | –0.14 *(0.10)* | [–0.36, 0.07] | 0.173 |
| Expected Satiating Quality | 0.44 *(0.11)* | [–0.20, 0.67] | 0.001 | 0.15 *(0.11)* | [–0.07, 0.36] | 0.171 |
| **Model** |  | | |  | | |
| R^2^/Adjusted R^2^ | 0.308/0.249 | | | 0.191/0.122 | | |

Notes: Effects are estimated using linear regression models. Expectation ratings are z-scored. DV: dependent variable; WTP: willingness to pay; B: unstandardized estimate; SE: standard error of the estimate; CI: confidence interval; n = 39.

Supplementary Table S6. Association between gender and claim effects on expected attributes, perceived taste pleasantness, and willingness to pay in Study 2

|  | **DV: Expected taste pleasantness** | | | **DV: Expected healthiness** | | | **DV: Expected satiating quality** | | |
| --- | --- | --- | --- | --- | --- | --- | --- | --- | --- |
| **Fixed effects** | **B (*SE*)** | **95% CI** | ***p*** | **B (SE)** | **95% CI** | ***p*** | **B (SE)** | **95% CI** | ***p*** |
| Intercept | –1.03 (*0.35*) | [–1.73, –0.32] | 0.01 | 1.08 (*0.22*) | [0.63, 1.53] | < 0.001 | 0.47 (*0.24*) | [–0.01, 0.96] | 0.06 |
| Gender  (1 = Man, 0 = Woman) | 0.38 (*0.48*) | [–0.61, 1.36] | 0.44 | –0-15 (*0.32*) | [–0.79, 0.48] | 0.63 | 0.15 (*0.33*) | [–0.53, 0.83] | 0.65 |
| **Model** |  |  |  |  |  |  |  |  |  |
| R2/R2 adjusted | 0.016/–0.011 | | | 0.007/–0.020 | | | 0.006/–0.021 | | |
|  | **DV: Perceived taste pleasantness** | | | **DV: WTP** | | |  |  |  |
| **Fixed effects** | **B (SE)** | **95% CI** | ***p*** | **B (SE)** | **95% CI** | ***p*** |  |  |  |
| Intercept | –0.04 (*0.12*) | [–0.29, 0.21] | 0.76 | 0.15 (*0.07*) | [0.01, 0.30] | 0.04 |  |  |  |
| Gender  (1 = Man, 0 = Woman) | 0.01 (*0.17*) | [–0.34, 0.36] | 0.95 | 0.001 (*0.10*) | [–0.20, 0.20] | 0.99 |  |  |  |
| **Model** |  |  |  |  |  |  |  |  |  |
| R2/R2 adjusted | 0.000/–0.027 | | | 0.000/–0.027 | | |  |  |  |

Notes: Effects are estimated using linear regression models. DV: dependent variable; B: unstandardized estimate; SE = standard error of the estimate; CI: confidence interval; n_Women_ = 19, n_Men_ = 20.

Supplementary Table S7. Effect of expectations and perceived taste pleasantness on the probability of preferring a drink with the “protein-rich” claim in Study 2

|  | **DV: Preferred a drink with the "protein–rich" nutrition claim** | | | | | |
| --- | --- | --- | --- | --- | --- | --- |
| **Fixed effects** | ***OR (SE)*** | **95% CI** | ***p*** | ***OR*** | **95% CI** | ***p*** |
| Intercept | 0.97 (*0.37*) | [0.45, 2.11] | 0.933 | 0.95 (*0.31*) | [0.50, 1.81] | 0.883 |
| Perceived Taste Pleasantness Difference | 3.04 (*1.71*) | [1.21, 11.63] | 0.048 |  |  |  |
| Expected Taste Pleasantness Difference | 0.77 (*0.31*) | [0.29, 1.62] | 0.521 |  |  |  |
| Expected Healthiness Difference | 2.29 (*1.04*) | [0.99, 6.13] | 0.070 |  |  |  |
| Expected Satiating Quality Difference | 1.48 (*0.75*) | [0.59, 4.39] | 0.434 |  |  |  |
| Taste Pleasantness Prediction Error | | |  | 1.38 (*0.50*) | [0.71, 3.16] | 0.378 |
| **Model** |  |  |  |  |  |  |
| R^2^ Tjur | 0.284 |  |  | 0.021 |  |  |

*Notes:* Effects are estimated using logistic regression models. R^2^ Tjur (a type of pseudo R^2^) indicates the distance between two means for the predicted values. The closer this value gets to 1, the clearer the separation between the predicted values for 0 and 1. Differences in expected and perceived attributes are *z*-scored. Differences in taste pleasantness prediction errors are entered in a separate model because as mentioned previously they are simply the difference between perceived and expected taste pleasantness and thus correlate highly with both these ratings. DV: dependent variable; *OR*: odds ratio; *SE* = standard error; CI: confidence interval; *n* = 39.

Supplementary Table S8. Small volume correction analyses results

|  |  |  |  |  | **MNI coordinates** | | |  |  |
| --- | --- | --- | --- | --- | --- | --- | --- | --- | --- |
|  |  | **k** | ***T*-value** | ***z*-value** | **x** | **y** | **z** | **Side** | **Region** |
| *Tasting drinks > Rinsing* | | | | | | | | | |
|  |  | 43 | 6.16 | 4.97 | 15 | –7 | 23 | R | Caudate |
|  |  | 52 | 6.10 | 4.93 | –18 | –19 | 20 | L | Caudate |
|  |  | 54 | 5.01 | 4.28 | –21 | 20 | –16 | L | Inferior Frontal Gyrus  (*pars orbitalis*) |
|  |  | 20 | 4.94 | 4.23 | 9 | 8 | 2 | R | Caudate |
| *Swallowing drinks > Swallowing rinse* | | | | | | | | | |
|  |  | 228 | 7.37 | 5.60 | –33 | 14 | 5 | L | Insula |
|  |  | 316 | 6.89 | 5.36 | 51 | 11 | 14 | R | Inferior Frontal Gyrus  (*pars Operculum*) |
|  |  | 27 | 5.53 | 4.47 | –9 | 23 | 29 | L | Anterior Cingulate Cortex |

*Notes:* Small volume correction was applied over a common mask including regions associated with taste and flavor processing at the whole-brain results thresholded at *p*_unc._ < 0.001. The table includes only peaks with *p*_FWE_ < 0.05 after small volume correction. Brain regions are labelled using the Anatomy and AAL3 toolboxes. L: left, R: right.

- - 1. Supplementary Figures

**Supplementary Figure S3. Common masks including regions of interest associated with taste (A.) and valuation (B.) processing used for small volume correction analyses. (A.)** To assess taste and flavor responses in the brain, we small volume corrected whole-brain results over a common mask including regions associated with taste and flavor processing. This mask includes bilateral orbitofrontal cortex, insula, frontal and rolandic operculum, anterior cingulate cortex, amygdala, caudate, putamen, pallidum, and thalamus. **(B.)** To assess claim effects on valuation, we small volume corrected whole-brain results over a common mask including regions previously associated with valuation. This mask includes left dorsolateral prefrontal cortex (shown at x = –48), bilateral nucleus accumbens (shown at y = 10), ventromedial prefrontal cortex (shown at z = –8) and left lateral orbitofrontal cortex (shown at z = –8). Regions of interest are overlayed on the ch2bet template. L: left; R: right.


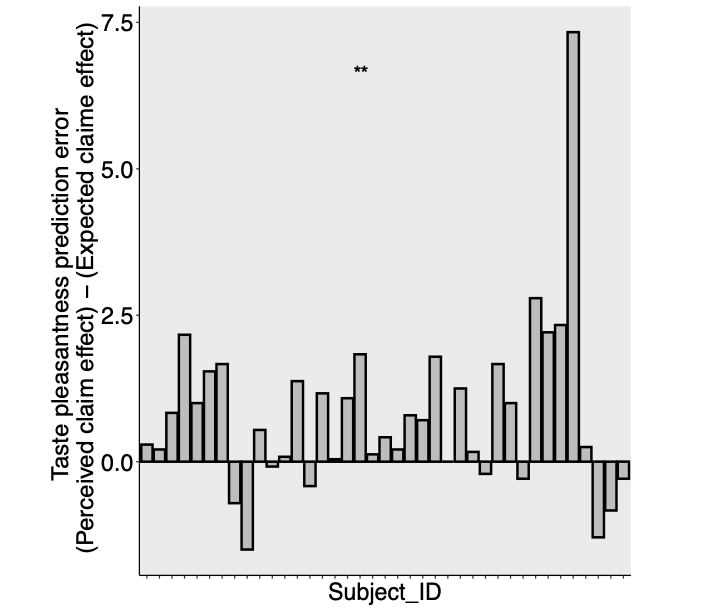


**Supplementary Figure S4. Taste pleasantness prediction errors in Study 2.** Prediction errors with regard to the claim effect on taste pleasantness. Participants perceived more claim advantage than expected (majority of prediction errors > 0; one sample *t*-test *t*_(38)_ = 3.40, *p* = 0.002); Gray bars are mean values for each participant. Ratings are pooled across both flavors. *n* = 39; ** *p* < 0.01.

**Supplementary Figure S5. Brain activations** **for tasting (A.) and swallowing (B.) drinks vs. rinsing solution.** Small volume correcting over common regions associated with taste processing revealed activation in bilateral caudate (green arrows in A.) and orbital part of frontal gyrus during tasting, and left insula, right operculum (green arrows in B.), and left anterior cingulate cortex during swallowing drinks. T-maps are overlayed on the ch2bet template. L: left; R: right; n = 33.

**Supplementary Figure S6.** **The midbrain cluster more active when tasting drinks without vs. with the “protein-rich” claim (shown in red) extends into the left nucleus accumbens (shown in blue) and to a small degree into the left pallidum (shown in green).** Images are overlayed on the ch2bet SPM template and are shown on coronal slices of 1 mm width from y = 1 to y = 7. The cluster in red survives p_FWE_ < 0.05 after small-volume-correction over regions commonly associated with valuation (n = 33). The anatomical left nucleus accumbens (blue) and left pallidum (green) were defined using the WFU Pickatlas toolbox for SPM. Clusters are overlayed on the ch2bet template. L: left; R: right.

REFERENCES

Bartoshuk, L. M. (1968). Water taste in man’. *Percept. Psychophys.* 3, 69–72. Available at: https://link.springer.com/content/pdf/10.3758/BF03212715.pdf [Accessed September 13, 2018].

Chamoun, E., Liu, A. A. S., Duizer, L. M., Darlington, G., Duncan, A. M., Haines, J., et al. (2019). Taste Sensitivity and Taste Preference Measures Are Correlated in Healthy Young Adults. *Chem. Senses* 44, 129–134. doi:10.1093/chemse/bjy082.

de Araujo, I. E. T., Kringelbach, M. L., Rolls, E. T., and McGlone, F. (2003). Human Cortical Responses to Water in the Mouth, and the Effects of Thirst. *J. Neurophysiol.* 90, 1865–1876. doi:10.1152/jn.00297.2003.

Esteban, O., Birman, D., Schaer, M., Koyejo, O. O., Poldrack, R. A., and Gorgolewski, K. J. (2017). MRIQC: Advancing the automatic prediction of image quality in MRI from unseen sites. *PLoS One* 12, e0184661. doi:10.1371/journal.pone.0184661.

Hardikar, S., Höchenberger, R., Villringer, A., and Ohla, K. (2017). Higher sensitivity to sweet and salty taste in obese compared to lean individuals. *Appetite* 111, 158–165. doi:10.1016/j.appet.2016.12.017.

Höchenberger, R., and Ohla, K. (2017). Rapid Estimation of Gustatory Sensitivity Thresholds with SIAM and QUEST. *Front. Psychol.* 8, 1–13. doi:10.3389/fpsyg.2017.00981.

Höchenberger, R., and Ohla, K. (2019). Repeatability of Taste Recognition Threshold Measurements with QUEST and Quick Yes–No. *Nutrients* 12, 24. doi:10.3390/nu12010024.

McLaren, D. G., Ries, M. L., Xu, G., and Johnson, S. C. (2012). A generalized form of context-dependent psychophysiological interactions (gPPI): A comparison to standard approaches. *Neuroimage* 61, 1277–1286. doi:10.1016/j.neuroimage.2012.03.068.

Seubert, J., Ohla, K., Yokomukai, Y., Kellermann, T., and Lundström, J. N. (2015). Superadditive opercular activation to food flavor is mediated by enhanced temporal and limbic coupling. *Hum. Brain Mapp.* 36, 1662–1676. doi:10.1002/hbm.22728.

Watson, A. B. (2017). QUEST+: A general multidimensional Bayesian adaptive psychometric method. *J. Vis.* 17, 10. doi:10.1167/17.3.10.
